# Broad substrate tolerance of tubulin tyrosine ligase enables one-step site-specific enzymatic protein labeling†
†The technology described in this manuscript is part of a pending patent application.
‡
‡Electronic supplementary information (ESI) available: Supporting figures, synthetic details, HPLC and MS spectra, ubiquitin labeling. See DOI: 10.1039/c7sc00574a



**DOI:** 10.1039/c7sc00574a

**Published:** 2017-03-20

**Authors:** Dominik Schumacher, Oliver Lemke, Jonas Helma, Lena Gerszonowicz, Verena Waller, Tina Stoschek, Patrick M. Durkin, Nediljko Budisa, Heinrich Leonhardt, Bettina G. Keller, Christian P. R. Hackenberger

**Affiliations:** a Department of Chemical-Biology , Leibniz-Institut für Molekulare Pharmakologie (FMP) , Robert-Rössle-Str. 10 , 13125 Berlin , Germany . Email: hackenbe@fmp-berlin.de; b Department of Chemistry , Humboldt Universität zu Berlin , Brook-Taylor-Strasse 2 , 12489 Berlin , Germany; c Department of Biology, Chemistry, Pharmacy , Freie Universität Berlin , Takustr. 3 , 14195 Berlin , Germany . Email: bettina.keller@fu-berlin.de; d Department of Biology II , Ludwig Maximilians Universität München and Center for Integrated Protein Science Munich , Großhadenerstr. 2 , 82152 Martinsried , Germany; e Department of Chemistry , TU Berlin , Müller-Breslau-Str. 10 , 10623 Berlin , Germany

## Abstract

The broad substrate tolerance of tubulin tyrosine ligase is the basic rationale behind its wide applicability for chemoenzymatic protein functionalization. In this context, we report that the wild-type enzyme enables ligation of various unnatural amino acids that are substantially bigger than and structurally unrelated to the natural substrate, tyrosine, without the need for extensive protein engineering. This unusual substrate flexibility is due to the fact that the enzyme's catalytic pocket forms an extended cavity during ligation, as confirmed by docking experiments and all-atom molecular dynamics simulations. This feature enabled one-step C-terminal biotinylation and fluorescent coumarin labeling of various functional proteins as demonstrated with ubiquitin, an antigen binding nanobody, and the apoptosis marker Annexin V. Its broad substrate tolerance establishes tubulin tyrosine ligase as a powerful tool for *in vitro* enzyme-mediated protein modification with single functional amino acids in a specific structural context.

## Abstract 2

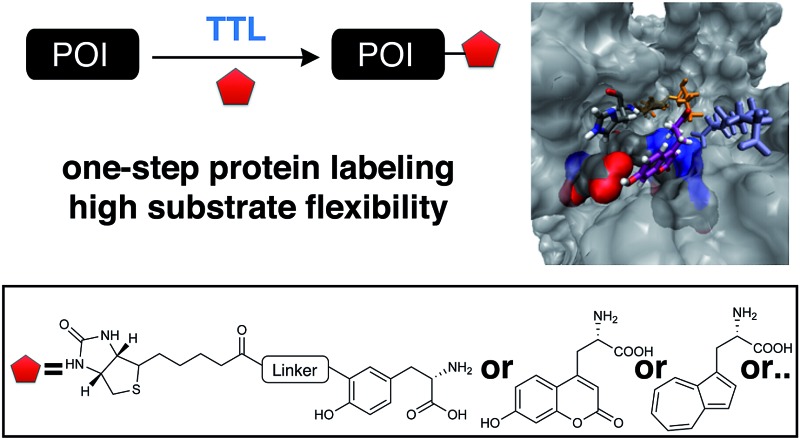
The broad substrate tolerance of tubulin tyrosine ligase enables its wide applicability for protein functionalization.

## Introduction

**Fig. 1:**
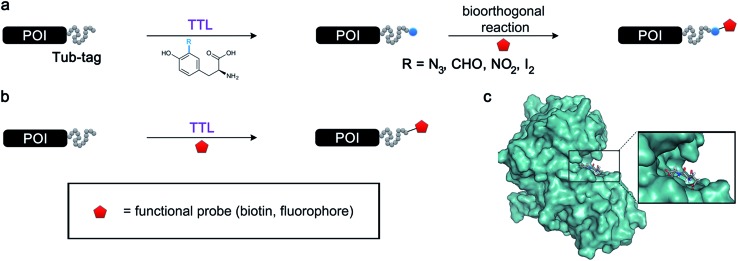
Protein functionalization by TTL. (a) Illustration of the two-step functionalization of proteins using Tub-tag labeling. A tyrosine derivative carrying a bioorthogonal handle at the *ortho*-position is incorporated into the C-terminal Tub-tag of a protein of choice. In the second step, a functional group (*e.g.* biotin or a fluorophore) is covalently attached to the protein by a bioorthogonal reaction. (b) One-step functionalization of proteins using an expanded substrate scope of TTL. Tyrosine derivatives that carry biotin (as shown) or a fluorophore are ligated to the proteins’ C-terminus by TTL. (c) Upon binding of ATP and α-tubulin, the enzymatic pocket of TTL forms a cavity, opening space for larger tyrosine derivatives (4IHJ). Structure modified from Prota *et al.* ^34^

The site-specific modification of biomolecules by chemical and/or molecular biology tools has become a standard tool in cellular and molecular biology as well as in the design of biopharmaceuticals.^1–5^ For instance, the fusion of fluorescent proteins^6–8^ and attachment of small organic fluorophores^9,10^ enable the precise localization of proteins within complex cellular systems. Newly arising technologies allow the controlled installation of post-translational modifications to proteins at predefined positions, giving an insight into regulatory processes within the complex proteome of an organism.^11–16^ Moreover, stabilizing peptides and proteins by the homogeneous attachment of polymer chains represents a milestone in the development of potent protein-based biomedicals.^17^ Ever since biotin ligase has been engineered to site-specifically modify peptides and proteins,^18,19^ the concept of repurposing naturally occurring enzymes for the labeling of biomolecules has gained ground and several valuable chemoenzymatic technologies have been developed.^20^ Prominent examples include Sortase A,^21–23^ phosphopantetheinyl transferase (PPTase),^24^ lipoic acid ligase,^25^ SpyCatcher/SpyTag^26^ and formylglycine-generating enzyme.^27^ These enzymes either allow the incorporation of bioorthogonal handles, which facilitates the subsequent site-specific attachment of a labelling probe, or they accept substrates that carry the probe in the first place.^28^ Nevertheless, enzymes have evolved as highly specific catalysts and thus often have in their natural form a rather narrow tolerance towards substrate modification, limiting their applicability for protein modifications. Enzyme engineering is a prominent approach to circumvent these constraints;^29^ however, this process can be quite elaborate and results in several enzyme variants, each capable of attaching one specific substrate. Enzymes with a broad substrate scope could minimize tedious enzyme engineering and therefore help expand the protein functionalization toolbox. We have recently introduced Tub-tag labeling as a general method for the site-specific C-terminal labeling of proteins.^30,31^ In this method, tubulin tyrosine ligase (TTL), an enzyme that plays an important role in the homeostasis of microtubules,^32^ is repurposed to attach tyrosine derivatives to any protein that carries a short peptide tag (the so-called Tub-tag, VDSVEGEGEEEGEE) at its C-terminus. Based on previous work that used α-tubulin as the substrate for the C-terminal attachment of simple *ortho*-substituted N_3_- or CHO-tyrosine derivatives,^33^ we combined TTL labeling with several well-established bioorthogonal reactions for a modular two-step protein modification strategy (Fig. 1a). Utilizing this chemoenzymatic approach, we could attach various fluorophores and biotin to recombinant antibodies and perform immunoprecipitaion (IP) experiments and super-resolution microscopy. TTL is a member of the ATP-grasp superfamily, suggesting that the enzyme catalyzes the C-terminal activation of its recognition sequence in an ATP-dependent manner.^35^ Various studies suggest that the canonical amino acid tyrosine is not the only substrate of TTL.^36^ Forty years ago, Arce *et al.* showed that in addition to tyrosine, phenylalanine and 3,4-dihydroxyphenylalanine were also incorporated at the C-terminus of α-tubulin when added to rat brain extract.^37^ In a later study, it was shown that phenylalanine, *p*-NH_2_-phenylalanine and *p*-N_3_-phenylalaine can act as moderate inhibitors of TTL.^38^ These studies gave the first hints towards phenylalanine and its derivatives being possible substrates for TTL and that the hydroxyl group of tyrosine might not be essential for enzyme activity. Nevertheless, there was no experimental proof that derivatives of phenylalanine were ligated to the C-terminus of α-tubulin by TTL. In contrast to that, *o*-NO_2_-tyrosine,^39^ *o*-F-tyrosine,^40^ *o*-I-tyrosine,^41^ *o*-CHO-tyrosine, *o*-NH_2_-tyrosine and *o*-N_3_-tyrosine^33^ were successfully ligated to the C-terminus of α-tubulin by TTL, suggesting that the enzyme has a certain substrate tolerance towards tyrosine derivatives substituted at its *ortho*-position. However, current literature claims that there does not appear to be enough substrate flexibility for TTL to accept bigger substrates or even fluorescent amino acids.^33^

Even though the structural basis for the binding of TTL to the C-terminus of α-tubulin and ATP has been described in detail, the reasons for the enzyme’s promiscuity towards modification of the tyrosine substrate are still unknown.^34,42^ Prota *et al.* recently used X-ray crystallography to show that the enzyme forms an extended cavity for substrate binding.^34^ Based on these findings, we hypothesized that the shape of the extended cavity of the wild-type enzyme might indeed enable TTL to ligate further amino acid derivatives to allow even larger substrates for TTL that are structurally unrelated to tyrosine. We envisioned that this unprecedented substrate promiscuity would potentially allow one-step labelling protocols and make a second chemical reaction unnecessary, as shown in Fig. 1b. Here, we describe our findings in discovering new functional substrates for TTL labeling and further support these experimental data by computational modelling, which provides a better picture to understand the broad substrate scope and binding mechanism of this enzymatic ligation reaction.

## Results and discussion

### Elucidating the enzyme’s substrate scope

We started our investigations by ligating phenylalanine (**1**) and 3,4-dihydroxyphenylalanine (**2**) to a carboxyfluorescein-labeled Tub-tag peptide **3**.^30^ We analyzed the reaction process after 5 h of incubation at 37 °C by UPLC-MS analysis and compared the results to the previously published ligation efficiencies of tyrosine (**4**) and its *ortho*-substituted derivatives (**5** and **6**) that are known to be substrates of TTL (Table 1).^30,31,34^ Moreover, to shed light on the role of tyrosine's hydroxyl group, we performed ligation experiments with propynyloxy-tyrosine (**7**) and *p*-N_3_-phenylalanine (**8**). While the unsubstituted compounds **1** and **4**, as well as the *ortho*-substituted tyrosine derivatives **2**, **5** and **6**, were ligated in high conversions of up to 100% within 5 h of incubation (Table 1), the enzyme activity dropped as soon as phenylalanine was substituted at the *para* position with an azide or propargyl ether (37% for ligation of **7** and 17% for ligation of **8**, Table 1). This observation indicates that steric hindrance or electrostatic effects limit the variability of phenylalanine or tyrosine at the *para* position. Based on the finding that TTL ligates amino acids other than tyrosine, we wanted to elucidate whether the substrate scope could be further expanded and incubated peptide **3** with other amino acids. While we did not observe any ligation for small polar amino acids (for instance see entry 8 and ESI Table 1†), and histidine (**S1**) was conjugated to a very limited extent (<5%, ESI Fig. 1 and 2‡), ligation of a number of hydrophobic amino acid derivatives was indeed possible (Table 1). Even though leucine (**10**) is ligated to a small extent (10%), substrates that contain aromatic structures are advantageous. Tryptophan (**11**) and tryptophan derivatives (**12–17**) are ligated by TTL in up to 75% conversion. In contrast, alanine (**18**), valine (**19**), isoleucine (**20**), the decarboxylated tyrosine derivative tyramine (**21**) and d-tyrosine (**22**) are not accepted. Moreover, *t*-Bu protection of tyrosine’s carboxylic acid (**23**) prevents ligation, and only incorporation of hydrolyzed tyrosine (**4**) is observed. This indicates that the carboxylic acid moiety is mandatory for ligation by TTL. The absence of an aromatic group drastically reduces the ligation efficiency and beta-branched aliphatic residues do not seem to be processed at all.

**Table 1:** Different compounds tested as substrates for TTL^*a*^

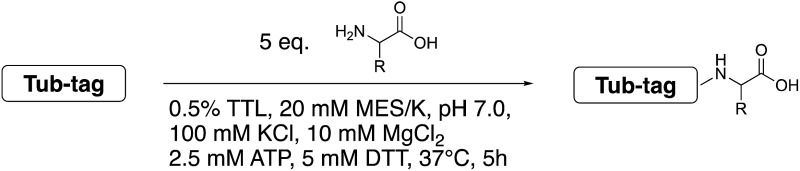									
Entry	Comp no.	Compound	d/l	Conversion [%]	Entry	Comp no.	Compound	d/l	Conversion [%]
1	**1**	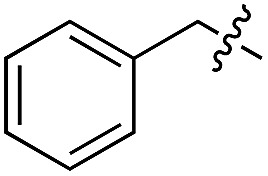	l	99	14	**15**	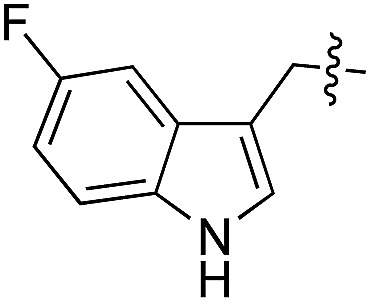	d/l	73
2	**2**	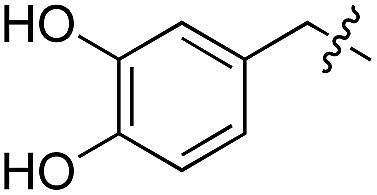	l	99	15	**16**	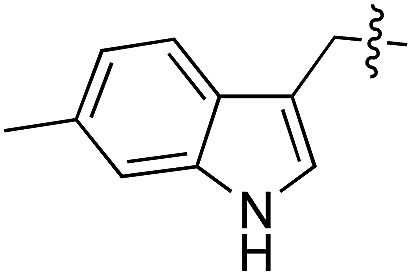	d/l	5
3	**4**	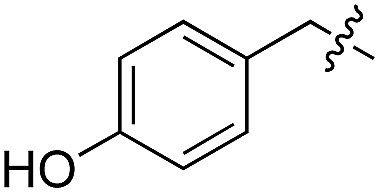	l	100 (ref. 34)	16	**17**	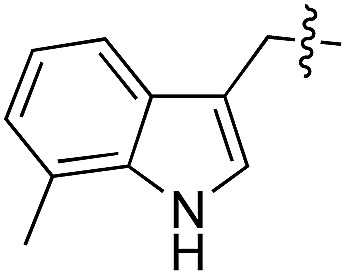	d/l	5
4	**5**	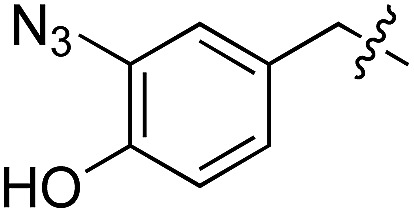	l	>90 (ref. 30)	17	**18**	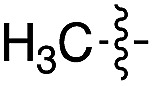	l	No conversion
5	**6**	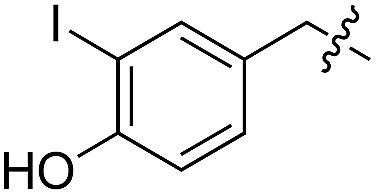	l	>90 (ref. 31)	18	**19**	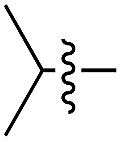	l	No conversion
6	**7**	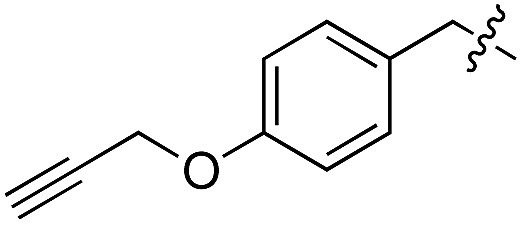	l	37	19	**20**	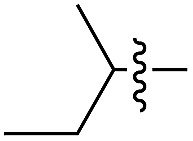	l	No conversion
7	**8**	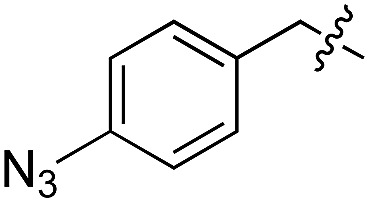	l	17	20	**21**	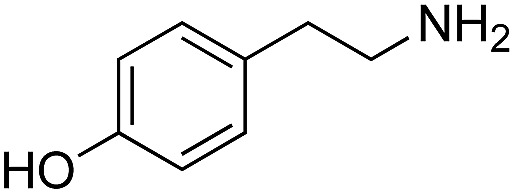	—	No conversion
8	**9**	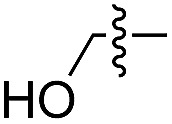	l	No conversion	21	**22**	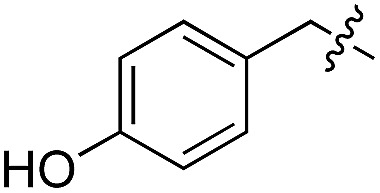	d	No conversion
9	**10**	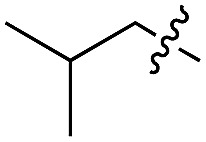	l	10	22	**23**	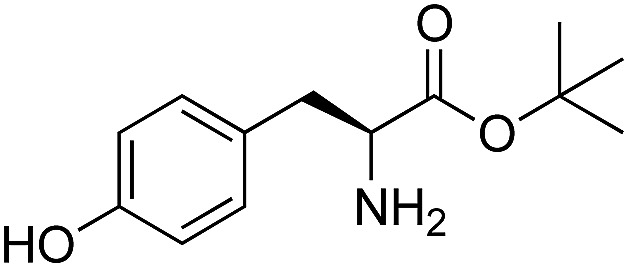	l	Hydrolysis
10	**11**	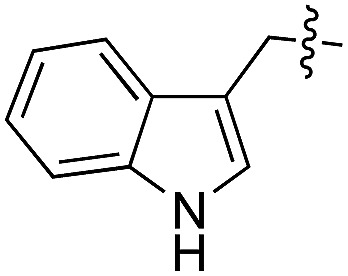	l	75	23	**24**	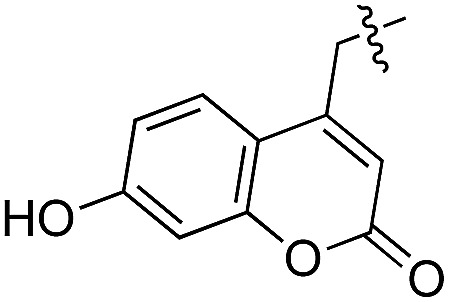	l	70
11	**12**	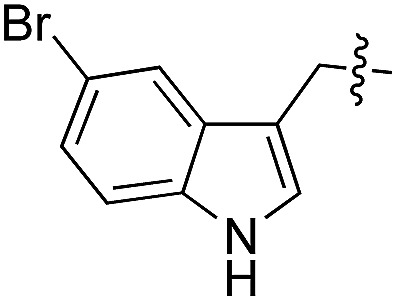	d/l	5	24	**25**	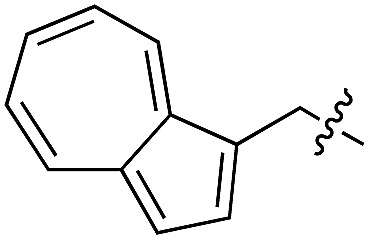	d/l	21
12	**13**	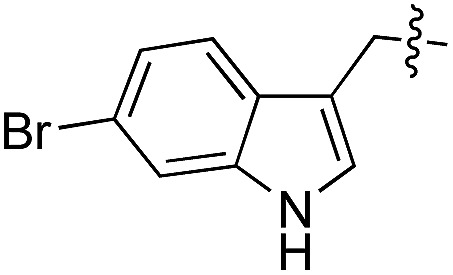	d/l	10	25	**26**	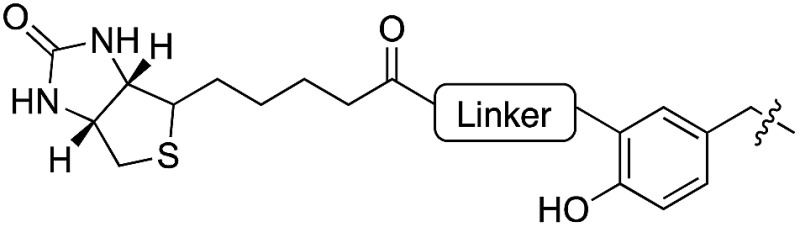	l	30
13	**14**	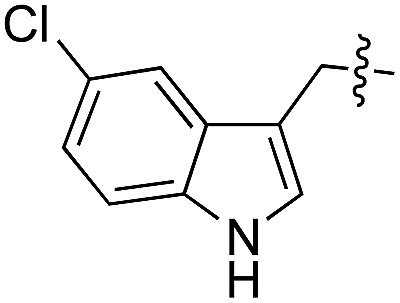	d/l	25		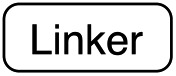	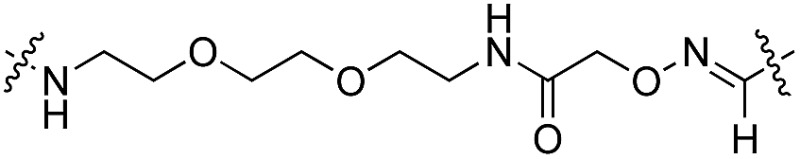		

^*a*^Tyrosination reactions were performed in a 250 μL solution consisting of 20 mM MES/K pH 7.0, 100 mM KCl, 10 mM MgCl_2_, 2.5 mM ATP, 1 mM L-amino acid derivatives or 2 mM racemic mixtures, 0.2 mM CF–Tub-tag, 1 μM TTL and 5 mM DTT. The mixture was incubated at 37 °C for 5 h and analysed by UPLC-MS. Quantities of substrate and product peptides were estimated from the corresponding peak areas in the TIC and the UV spectra. UV and MS spectra are shown in ESI Fig. 1–3. d/l: racemic mixture of amino acid applied at 2 mM concentration. l, d: l- or d-isomer of amino acid applied at 1 mM concentration.

Based on these observations, we tested TTL activity with functionally interesting aromatic amino acids. Thereby, we were able to demonstrate that the fluorescent coumarin amino acid (**24**)^43^ as well as β-(1-azulenyl)-l-alanine (**25**)^44^ was ligated by the enzyme (70% for **24** and 21% for **25**). In this context, the non-benzenoid aromatic amino acid **25** is a particularly interesting non-environmentally sensitive fluorescent probe, which has been demonstrated to bear attractive spectroscopic properties.^45,46^ While a related coumarin amino acid has been incorporated into proteins by amber suppression and enzymatic ligation, it is worth noting that Tub-tag labeling capitalizes on the first successful site-specific chemoenzymatic incorporation of an azulenyl amino acid into proteins.^47,48^

To further elucidate the enzyme’s ability to ligate even larger substrates, we synthesized biotinylated tyrosine **26** by an oxime forming reaction of 3-formyl-tyrosine (**S3**) and a biotinylated hydroxylamine (**S12**, see ESI for details‡). A short ethylene glycol linker was used to enable substrate flexibility and to allow the biotin moiety to protrude from the enzyme’s cavity (Table 1). After 5 h of incubation we indeed observed a conversion of 30%. These results indicate that the extended cavity of the active site of the enzyme results in a unique substrate tolerance of the ligase. Taken together, the incorporation of structurally and functionally interesting amino acids **24–26** underlines the power of TTL-mediated ligations for the one-step site-specific labelling of proteins.

### Computational studies

**Fig. 2:**
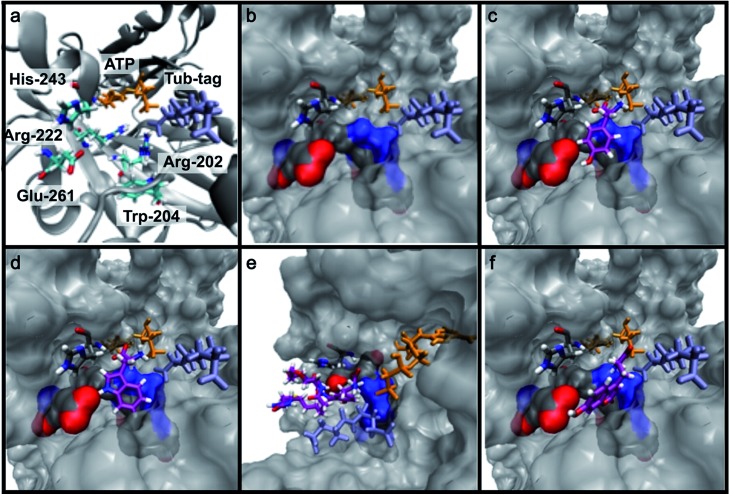
(a) Depiction of the binding pocket highlighting the characteristic features: ACP/ATP (orange), Tub-tag (violet-gray), as well as important amino acids. (b) Representation of the active site as a surface plot depicting the main properties: negatively charged oxygen atoms (red, glutamic acid-261), positively charged amine groups (blue, arginine-202, arginine-222), as well as the π-system of histidine-243. Docking conformations (purple) of (c) tyrosine (**4**), (d) tryptophan (**11**), (e) biotinylated tyrosine **26** and (f) coumarin derivative (**24**).

To get further insights into the substrate scope of TTL and to rationalize the observed differences in ligation rates as presented in Table 1, docking studies using AutoDockTools^49^ were performed on various substrates. As a reference system, the binding pose of the original substrate tyrosine (**4**) was investigated. Tyrosine is stabilized in the binding pocket by five main features (Fig. 2a–c).

First, glutamic acid-261 provides a hydrogen bond acceptor for the hydroxyl residue. Second, T-shaped π-stacking interactions with histidine-243 are observed. As a third characteristic, a small positively charged pocket next to the *ortho*-position of tyrosine is formed by arginine-202 and tryptophan-204. Fourth, arginine-222 stabilizes the carboxyl group of tyrosine. Together, these properties stabilize the binding of tyrosine in a reactive position and explain the fast ligation rates of tyrosine that have been shown before.^42^ Finally, the fifth important feature is the extended cavity towards the enzyme's surroundings (Fig. 1).

In addition to the docking studies, an all-atom molecular dynamics simulation of tyrosine (**4**) bound to TTL was performed using GROMACS 5.0.2.^50^ The simulation showed that the cavity is stable in solution. It also revealed a rotation of the aromatic ring of tyrosine along the ring axis. During the entire simulation, tyrosine stayed in contact with histidine-243, resulting in permanent π-stacking interactions, either in a T-shape or in a parallel-displaced conformation. This observation indicates that the π-interaction with histidine-243 might be the most important property of the binding pocket.

This model explains the high conversion rates observed for the other canonical amino acids phenylalanine (**1**) and tryptophan (**11**) that can take advantage of these π-interactions based on their aromatic ring structures. Especially in the case of tryptophan (**11**), similar binding behavior as for tyrosine is observed. The benzyl ring points towards the positively charged pocket next to the *ortho*-position of tyrosine and the pyrrole ring forms a hydrogen bond with glutamic acid-261 (Fig. 2d). However, no free rotation of the indole system could be observed in an all-atom molecular dynamics simulation, which is the first hint towards the limitation of the pocket size. The fact that hydrophobic amino acids that lack aromatic structures show limited (leucine (**10**)) or no (substrates **18–20**) ligation to peptide **3** further substantiates the importance of the π-stacking interactions for efficient substrate ligation by the enzyme.

Next we investigated the binding interactions of tyrosine derivatives carrying a functional group at the *ortho*-position (**5–6**), similar to the derivatives used in the high-yielding two-step labeling of proteins.^30^ The docking studies of these derivatives showed that this reactivity can be traced back to three binding modes. For small substituents such as fluorine and chlorine that have a negative partial charge, the binding behavior is similar to that of tyrosine (**4**) and the substituent points towards the small positively charged pocket formed by arginine-202 and tryptophan-204 (ESI Fig. 4‡). For medium-sized substituents which carry a negative partial charge, like iodides and azides, the interaction with the positively charged pocket is still in place, but the ring is rotated by approx. 90° into a parallel-displaced π-stacking interaction (ESI Fig. 4‡). For large substituents, like the biotinylated substrate **26**, the ring is rotated such that the biotin points out of the binding pocket (Fig. 2c). This is possible because of the spacious cavity of the enzyme's active site. Besides this ring rotation, no changes in the binding behavior compared to natural tyrosine are observed, explaining the high conversion rates detected for such compounds. For all derivatives, π-stacking interactions with histidine-243 as well as hydrogen-bond formation with glutamic acid-261 are conserved.

As shown for compounds **7** and **8**, derivatization at the *para*-position of the aromatic ring of tyrosine and phenylalanine is less favored. The reason for this can be found in the restricted pocket size of the catalytic site close to the hydroxyl group of tyrosine. Glutamic acid-261 and alanine-339 as well as phenylalanine-263 block the elongation of the substrate at the *para*-position, leading to significant changes in the binding mode and to strained binding conformations (ESI Fig. 4f and g‡). Next, we investigated the impact of tryptophan derivatization at indole-position 5 (**12**, **14**, **15**), 6 (**13**, **16**) or 7 (**17**) on the binding mode. For all derivatives, lower conversion rates were observed compared to unsubstituted tryptophan (**11**, Table 1). As the indole system of tryptophan is already located in the small pocket formed by arginine-202 and tryptophan-204, any extension of the indole system induces steric clashes, which lead to different binding modes than for tryptophan (**11**). In addition, large rotations along the ring plane, as seen for tyrosine, were not observed for tryptophan (tested by all-atom molecular dynamics simulation). However, for small substituents in position 5, such as fluorine (**15**), a conversion rate comparable to that for unmodified tryptophan (**11**) is obtained. The high conversion rate of **15** can be explained through a slight rotation of tryptophan, pointing the partially negatively charged fluorine towards the positively charged pocket (arginine-202, ESI Fig. 4h‡). This rate decreases as the substituent size increases, because the electrostatic effect competes with the size of the substituent, as seen by the conversion rates of **12**, **14** and **15** in Table 1. For substitutions at positions 6 or 7, a completely changed substrate binding is observed. The indole system of the tryptophan flips, resulting in a new binding mode no longer comparable to tyrosine binding, and additionally it loses the hydrogen-bond interaction with glutamic acid-261 (ESI Fig. 4h and i‡).

As shown in Table 1, aside from canonical amino acids and their derivatives, the TTL can also ligate non-natural amino acids with aromatic side chains, such as the coumarin derivative **24**. Docking studies revealed that coumarin **24** takes advantage of all the properties of the active site of TTL (Fig. 2f). The hydroxyl group of **24** forms a hydrogen bond to glutamic acid-261, and π-stacking interactions between histidine-243, tryptophan-204 and the aromatic system of **24** are observed. Moreover, the lactone moiety of **24** provides negative partial charges pointing towards arginine-202 and arginine-222, further stabilizing the binding mode within the catalytic pocket.

### One-step fluorescent labeling of functional proteins

**Fig. 3:**
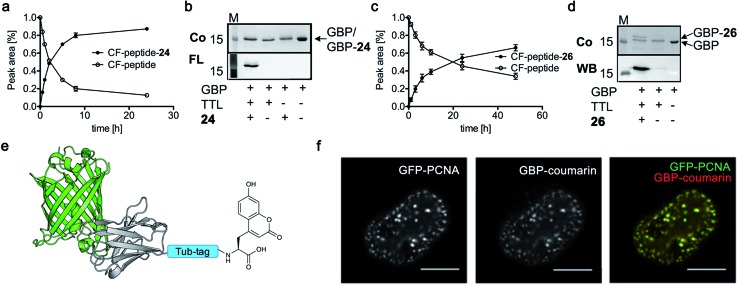
(a and c) C-terminal addition of coumarin **24** (a) and biotin **26** (c) to Tub-tag peptide **3**. UPLC-MS traces were taken at different time points of the TTL reaction and quantitation of substrate and product was performed through peak integration as described before.^30^ The mean values and standard deviations (SDs) of three replicate reactions are shown. (b and d) Incorporation of **24** (b) and **26** (d) into the C-terminus of GBP after six (**24**, b) or twenty hours (**26**, d) of incubation at 37 °C. The gel shift in SDS-PAGE and the fluorescence signal, as well as the signal in the Strep-HRP Western blot, show ligation of **24** and **26** to the C-terminus of Tub-tagged GBP (conversion of the TTL reaction for biotin **26** is determined by SDS-PAGE analysis based on the ratio of ligated and unligated nanobody. M: marker, Co: Coomassie staining, FL: fluorescence detection, 320 nm. WB: Western Blot, Strep-HRP). (e) Schematic illustration of coumarin **24** functionalized GBP binding its antigen GFP (PDB code: ; 3K1K). (f) Immunofluorescence of GFP-PCNA with a GFP-specific nanobody (GBP), functionalized with coumarin **24** *via* Tub-tag-mediated coumarin incorporation. Scale bar is 5 μm.

Based on the reactivity observed in Table 1 and the docking experiments, we further analyzed the reaction of the functionally interesting compounds **7**, **11**, **15**, **24** and **26**, and quantified product formation at different time points (Fig. 3a and c for **24** and **26**, ESI Fig. 5–7‡ for **7**, **11** and **15**). Indoles **11** or **15**, as well as coumarin **24**, have higher initial reaction rates compared to propynyloxy-tyrosine (**7**) and reach high conversions of over 80% within 8–10 hours. For the biotinylated tyrosine derivative **26**, we observed a conversion of 66% after 48 hours (Fig. 3c). It should be noted that full product conversion was not observed during the ligation of additional TTL substrates under the given reaction conditions. Further studies to address this observation, in particular to evaluate the stability of the enzyme under prolonged reaction times, are currently underway.

Encouraged by these results, we wanted to investigate whether this unique reactivity holds true for the labeling of functional proteins. We therefore probed the one-step Tub-tag labeling with biotin derivative **26** and coumarin **24**. We started by incubating **24** with the eukaryotic protein ubiquitin, equipped with a C-terminal 14 amino acid Tub-tag sequence (VDSVEGEGEEEGEE).^51,52^ Ligations were performed using a TTL/Ub ratio of 1 : 10 at 37 °C for up to 6 hours. SDS-PAGE and in-gel fluorescence revealed the fluorescent labeling of ubiquitin with coumarin **24** (ESI Fig. 8‡).

Subsequently, we successfully applied one-step Tub-tag fluorescent labeling with coumarin **24** using the same conditions to a GFP binding nanobody, termed GBP (Fig. 3b and ESI Fig. 9‡). In addition, we applied biotin substrate **26** for the site-specific one-step biotin attachment to GBP. SDS-PAGE and Western blot analysis revealed that 40% of GBP was converted within twenty hours of incubation at 37 °C using a GBP : TTL ratio of 5 : 1 (Fig. 3c).

Nanobodies are small (∼13 kDa) and stable antibody fragments derived from Camelidae that have versatile uses in biochemistry and cell biology.^53,54^ Most of the time, nanobodies intended for imaging experiments are labeled by genetic fusion to fluorescent proteins.^8^ However, recent studies showed that the attachment of small organic fluorophores and probes increases their potential as widely applicable tools for molecular imaging and cell biology.^55–57^

To test whether one-step functionalized GBP (GBP-coumarin) can be applied for this purpose, we stained cells expressing three GFP-fusion proteins with specific localization inside the cell. Upon incubation with GBP-coumarin we found colocalizing focal signals with GFP-PCNA, a homotrimeric protein complex that serves as a loading platform for DNA-binding and -modifying factors (Fig. 3e). GFP-Dmnt1, a DNA methyltransferase enriched at sites of DNA replication during the S-phase, was visualized as well (ESI Fig. 10a‡). Moreover, we successfully stained cells expressing GFP-lamin, a structural protein that envelops the cell nucleus (ESI Fig. 10b‡).

**Fig. 4:**
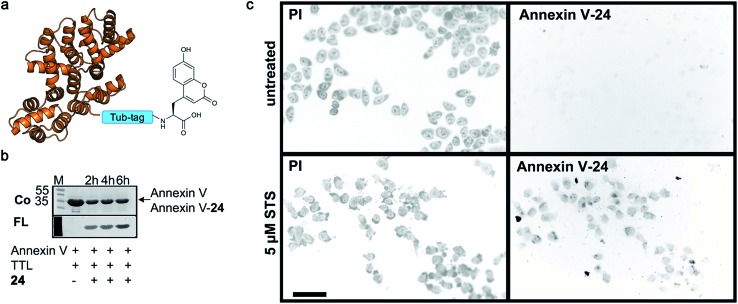
Detection of apoptotic cells with Annexin V-coumarin. (a) Schematic illustration of Annexin V functionalized with coumarin **24** (PDB code: ; 1SAV) (b) TTL-mediated incorporation of **24** into the C-terminus of Annexin V at 37 °C. The fluorescence signal shows successful ligation of **24** (M: marker, Co: Coomassie staining, FL: fluorescence detection, 320 nm). Staurosporine-treated (5 μM; lower panel) and untreated cells (upper panel) were stained with Annexin V-**24**, generated *via* Tub-tag-mediated functionalization and counterstained with propidium iodide. Scale bar is 50 μm.

Finally, we labeled Tub-tagged Annexin V with coumarin **24** (ESI Fig. 11‡). Annexin V is an endogenous human protein used as a marker for apoptosis. Annexin V strongly binds to phosphatidylserine, a phospholipid present in the cell membrane that flips from the cytosolic to the extracellular surface upon apoptosis, during programmed cell death.^58^ A variety of chemically modified Annexin V probes have been synthesized and some are commercially available.^59^ However, most of the markers available are heterogeneous mixtures conjugated using unspecific lysine chemistry. For *in vivo* use of Annexin V, the controlled attachment of a defined number of probes is desirable.^60^ After chemical induction of apoptosis with 5 μM staurosporine, apoptotic cells were visualized with coumarin-labeled Annexin V (Fig. 4), with comparable quality to a commercial probe (ESI Fig. 12‡), proposing a broad applicability of TTL-mediated site-specific coumarin-labeling of recombinant proteins for analytical purposes.

## Conclusion and perspective

We discovered that the substrate tolerance of wild-type tubulin tyrosine ligase is much broader than initially thought. This enabled us to ligate a variety of natural as well as unnatural amino acids not structurally related to the native substrate. Besides various tryptophan analogs, we could demonstrate the one-step enzymatic incorporation of functionally diverse azulenyl-, coumarin- and biotin-containing amino acids into peptides. Docking studies and all-atom molecular dynamics simulations revealed important insights into the enzymatic mechanism of TTL, showing high flexibility within the enzyme's catalytic pocket and the formation of an expanded cavity. These unique features of TTL substrate binding modes enabled us to broaden the scope of Tub-tag labeling to the one-step site-specific biotin- and fluorescent labeling of proteins, as demonstrated for the GFP-binding nanobody GBP, ubiquitin and the apoptosis marker Annexin V.

The unexpected broad substrate tolerance of TTL reported herein could be further enhanced by guided evolution protocols. In this way, we envision one-pot TTL-mediated ligation as a useful supplementary method for widespread site-specific ribosomal-mediated incorporation of unnatural amino acids into peptides.

## Conflicts of interest

The authors declare competing financial interests: the technology described in the manuscript is part of a pending patent application.
