# Supplementary material for: Broad substrate tolerance of tubulin tyrosine ligase enables one-step site-specific enzymatic protein labeling
Source: Chem Sci. 2017 Mar 20;8(5):3471–8. doi: 10.1039/c7sc00574a (PMC5418632; doi:10.1039/c7sc00574a)
Supplement: Supplementary file 1 [file SC-008-C7SC00574A-s001.pdf]

Electronic Supplementary Material (ESI) for Chemical Science.
This journal is © The Royal Society of Chemistry 2017

Supporting Information

Broad substrate tolerance of tubulin tyrosine ligase enables one-step site-specific enzymatic protein labeling

Dominik Schumacher,[a,b] Oliver Lemke,[c] Jonas Helma,[d] Lena Gerszonowicz,[b] Verena Waller,[d] Tina Stoschek,[d] Patrick A. Durkin,[e] Nediljko Budisa,[e] Heinrich Leonhardt,[d] Bettina G. Keller,[c,*] Christian P.R. Hackenberger[a,b,*]

1. SUPPLEMENTARY RESULTS 1
1.1. UPLC UV spectra 1
1.2 UPLC-MS spectra 2
1.3 Docking studies 4
1.4 Ligation of 7 to peptide 3 5
1.5 Ligation of 10 to peptide 3 6
1.6 Ligation of 14 to peptide 3 7
1.7 Coumarin labeling of Ubiquitin-Tub-tag 8
1.8 Coumarin labeling of GBP4-Tub-tag 9
1.9 Immunofluorescence of GFP fusion proteins 10
1.10 Coumarin labeling of Annexin V-Tub-tag 11
1.11 Detection of apoptotic cells with Annexin V_Coumarin 12
2. Material and Methods 13
2.1 General Information 13
2.2 Experimental Procedures 13
2.2.1 TTL expression and purification 13
2.2.2 Determination of TTL substrate scope using CF–Tub-tag peptide 14
2.2.3 GBP–Tub-tag expression and purification 14
2.2.4 Ubiquitin-Tub-tag expression and purification 14
2.2.5 Annexin V-Tub-tag expression and purification 14
2.2.6 Ligation of coumarin-derivative 24 to ubiquitin 14
2.2.7 Ligation of coumarin-derivative 24 to GBP 15
2.2.8 Ligation of coumarin-derivative 24 to Annexin V 15
2.2.9 Immunofluorescence for confocal microscopy 15
2.2.10 Annexin V staining 15
2.2.11 Microscopy 15
2.2.12 Docking studies 15
2.2.13 Molecular dynamics simulations 15
2.3. Chemical Synthesis 16
2.3.1 Synthesis of 3-formyl-*L*-tyrosine (S3) 16
2.3.2 Synthesis of Tyr(*o*-propargyl) (7) 17
2.3.3 Synthesis of (1S)-1-Carboxy-2-(7-hydroxy-2-oxo-2H-chromen-4-yl)ethyl ammonium trifluoroacetate (24) 18
2.3.4 Synthesis of Azuelenyl-alanine 25 20
2.3.4 Synthesis of tyrosine-biotin 26 23
2.4 Synthesis of CF–Tub-tag peptide 3 26
3. NMR Spectra of S3, 7, 24 and 26 27
3.1 3-formyl-*L*-tyrosine (2) 27
3.5 Tyr(*o*-propargyl) 7 28
3.5 Coumarin 24 29
3.5 Tyrosine biotin 26 30
4. References 31

1. SUPPLEMENTARY RESULTS

1.1. UPLC UV spectra

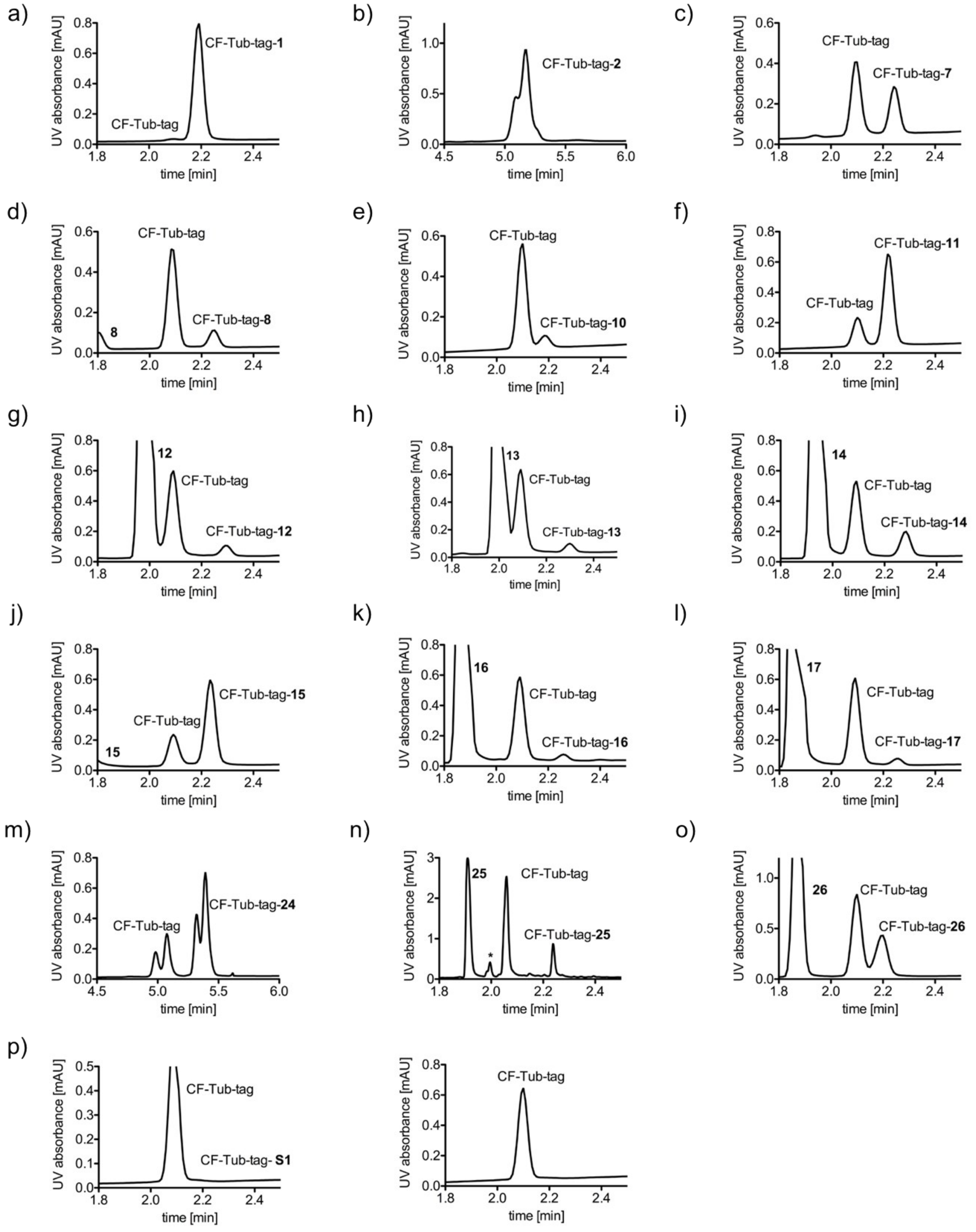

Supplementary Figure 1. Expanded substrate scope of TTL. UPLC UV-traces (220 nm) showing the ligation of various new substrates of TTL after five hours of incubation. (a-o) to the CF-Tub-tag peptide Carboxyfluorescein-VDSVEGEGEEEGEE (**3**, p) after five hours of incubation. (a) phenylalanine (**1**), (b) 3,4-dihydroxyphenylalanine (**2**) (c) ethynyloxy-tyrosine (**7**), (d) *p*-N_3-phenylalanine (**8**), (e) leucine (**10**), (f) tryptophan (**11**), (g) 5-Br-tryptophan (**12**), (h) 6-Br-tryptophan (**13**), (i) 5-Cl-tryptophan (**14**), (j) 5-Fl-tryptophan (**15**), (k) 6-methyl-tryptophan (**16**), (l) 7-methyl-tryptophan (**14**), (m) coumarin derivative **24**, (n) ß-(1-azulenyl)-L-alanine (**25**), * impurity from amino acid, (o) biotin-tyrosine (**26**), (p) histidine (**S1**), (q) peptide **3.** (a,c-l, n-q) UPLC method B, (b and m) UPLC method BII.

1.2 UPLC-MS spectra

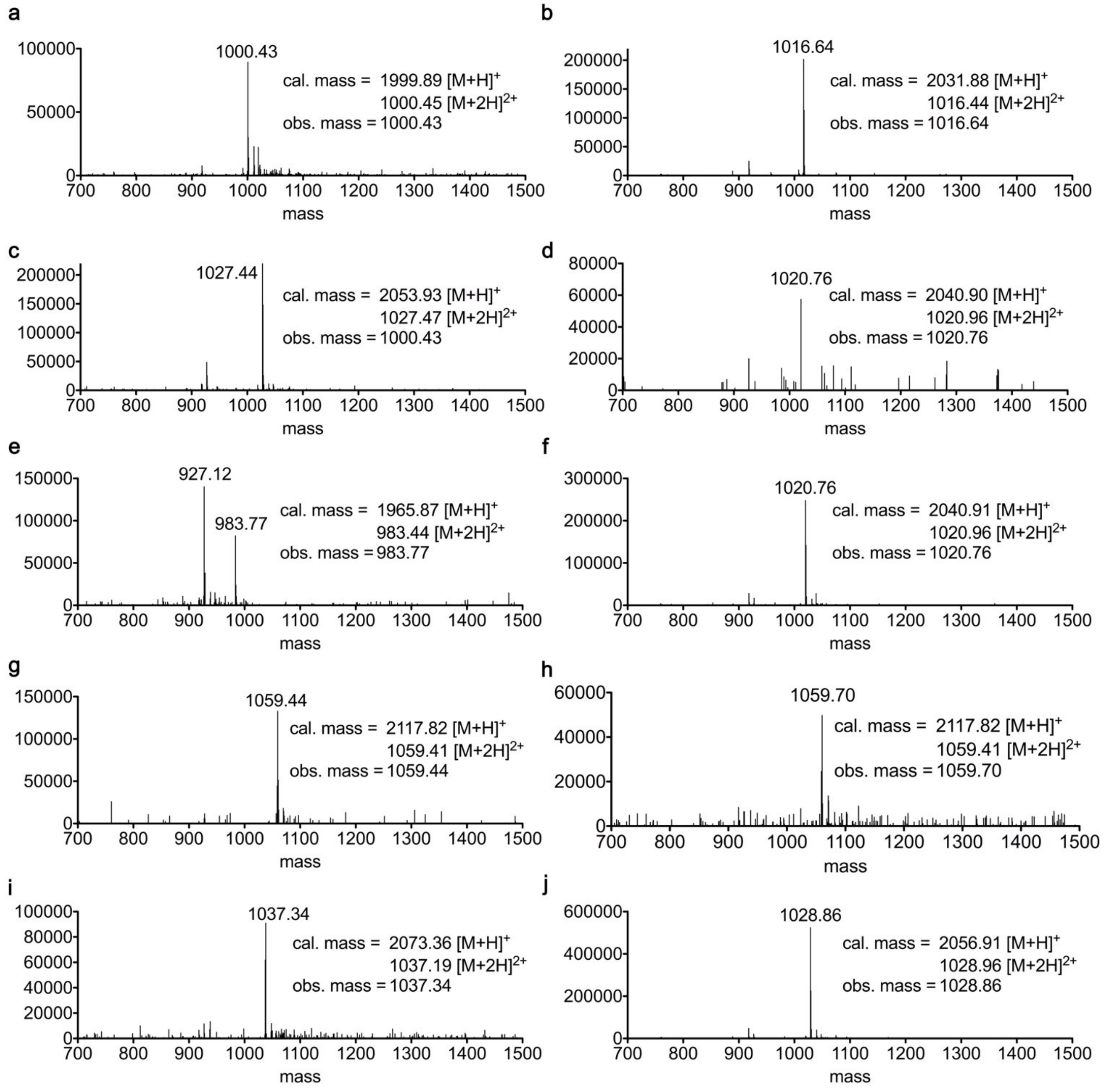

Supplementary Figure 2. Expanded substrate scope of TTL. MS spectra showing the ligation product of various new substrates of TTL after five hours of incubation. (a) phenylalanine (**1**), (b) 3,4-dihydroxyphenylalanine (**2**) (c) ethynyloxy-tyrosine (**7**), (d) *p*-N_3-phenylalanine (**8**), (e) leucine (**10**), (f) tryptophan (**11**), (g) 5-Br-tryptophan (**12**), (h) 6-Br-tryptophan (**13**), (i) 5-Cl-tryptophan (**14**), (j) 5-Fl-tryptophan.

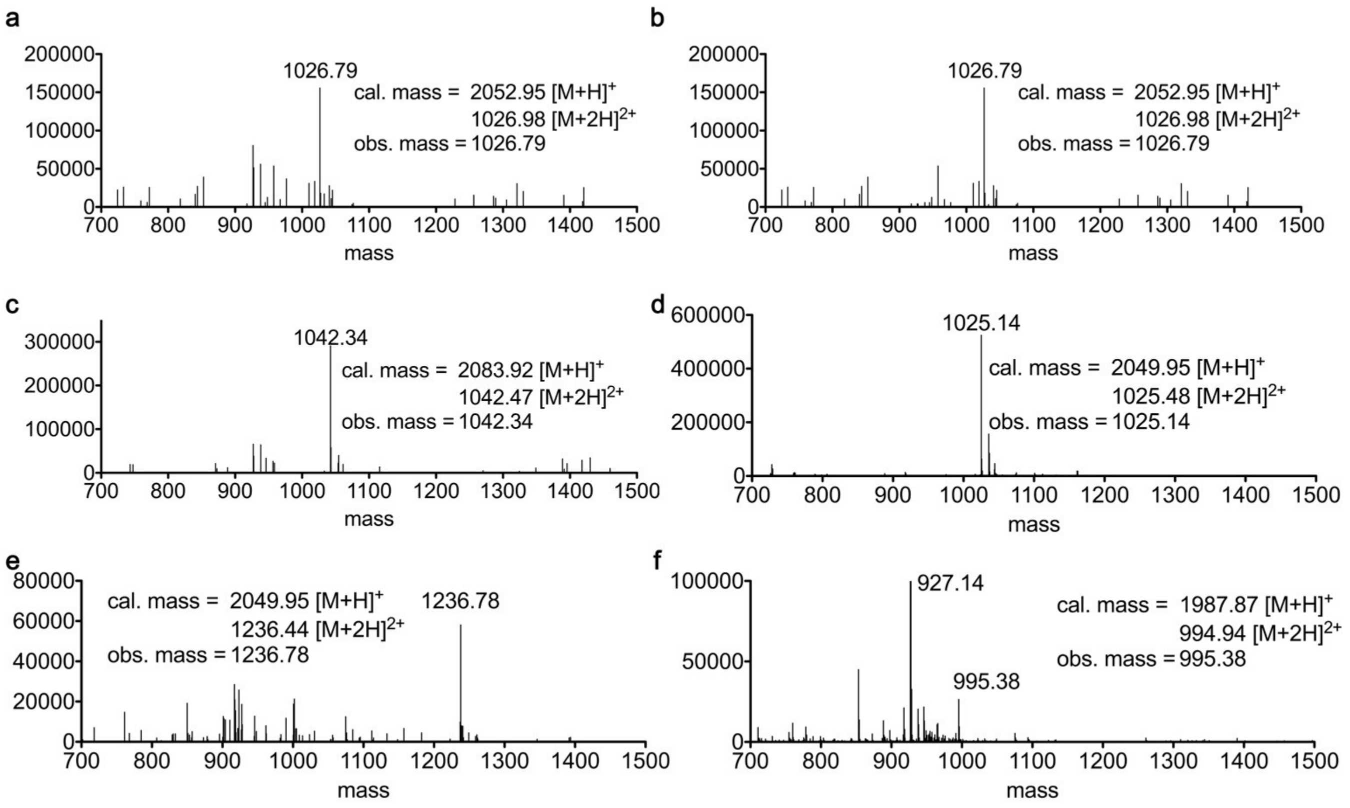

Supplementary Figure 3. Expanded substrate scope of TTL. MS spectra showing the ligation product of various new substrates of TTL after five hours of incubation. (a) 6-methyl-tryptophan (**16**), (b) 7-methyl-tryptophan (**14**), (c) coumarin derivative **24**, (d) ß-(1-azulenyl)-L-alanine (**25**), (e) biotin-tyrosine (**26**), (f) histidine (**S1**).

Supplementary Table 1. Additional amino acids tested for Tub-tag labeling that are not ligated by the TTL.

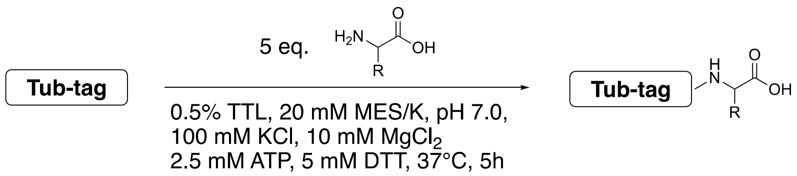

Entry	Comp No.	Compound	D/L	Entry	Comp No.	Compound	D/L
1	**S2**	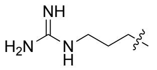	L	7	**S8**	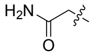	L
2	**S3**	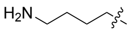	L	8	**S9**	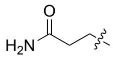	L
3	**S4**	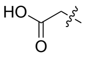	L	9	**S10**	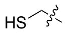	L
4	**S5**	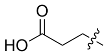	L	10	**S11**	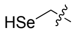	L
5	**S6**	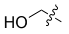	L	11	**S12**	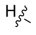	L
6	**S7**	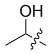	L	12	**S13**	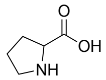	L

Table Legend: Tyrosination reactions were performed in a 250 µL solution consisting of 20 mM MES/K pH 7.0, 100 mM KCl, 10 mM $MgCl_2$, 2.5 mM ATP, 1 mM L amino acid derivative or 2 mM racemic mixtures, 0.2 mM CF–Tub-tag, 1 µM TTL and 5 mM DTT. The mixture was incubated at 37 °C for 5 h and analysed by UPLC-MS.

1.3 Docking studies

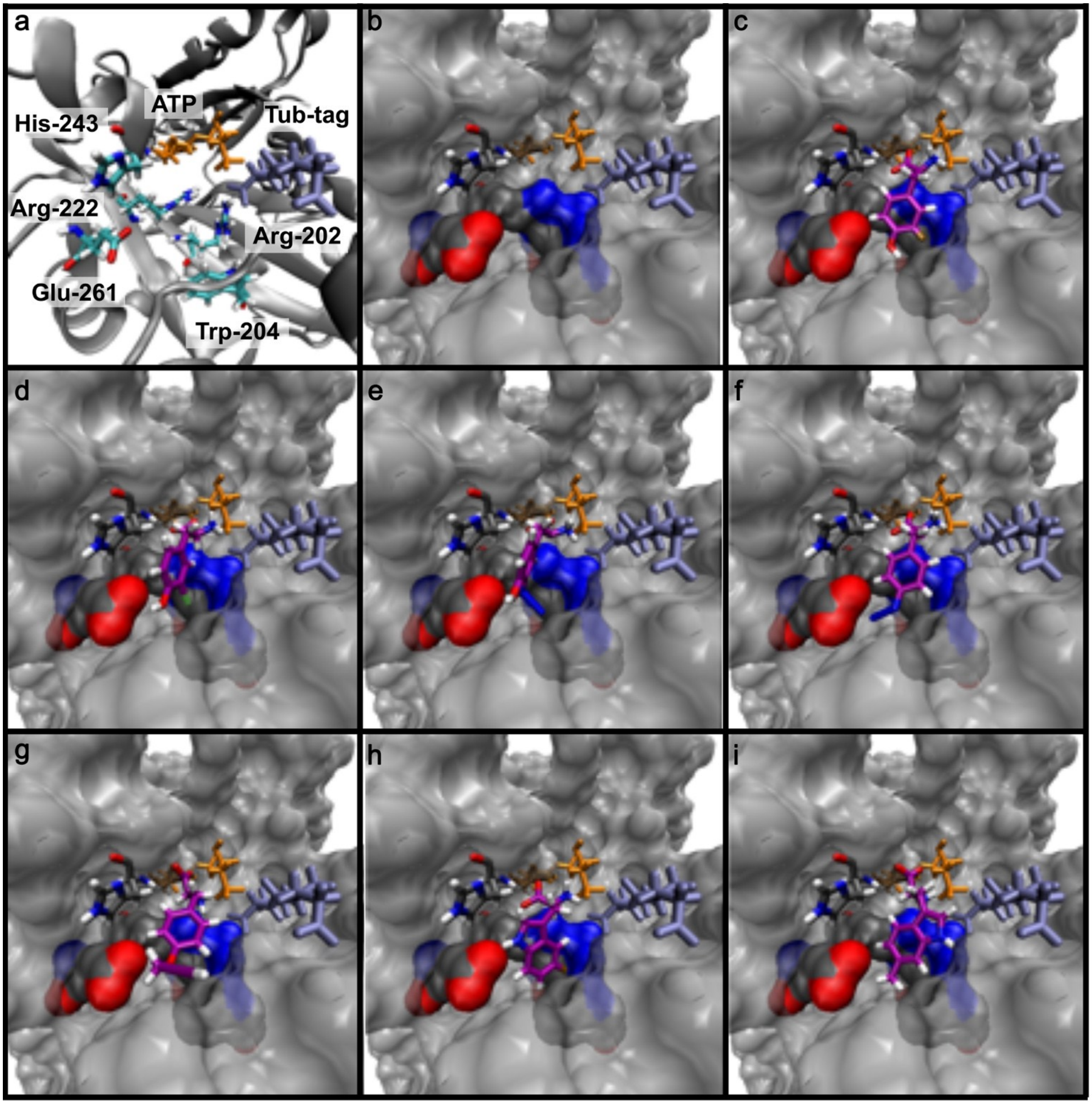

Supplementary Figure 4. (a) Depiction of the binding pocket highlighting the characteristic features: ACP/ATP (orange), Tub-Tag (violet-gray) as well as important amino acids. (b) Representation of the active site as a surface plot depicting the main properties: negatively charged oxigens (red), positively charged amines (blue) as well as the π-system of histidine-243. Docking conformation (purple) of (c) *o*-Cl-tyrosine, (d) *o*-I-tyrosine, (e) *o*-N_3-tyrosine (**5**), (f) *p*-N_3-phenylalanine (**8**) and (g) ethynyloxy-tyrosine (**7**) as well as (h) 5-F-tryptophan and (i) 6-methyl-tryptophan (**16**).

1.4 Ligation of 7 to peptide 3

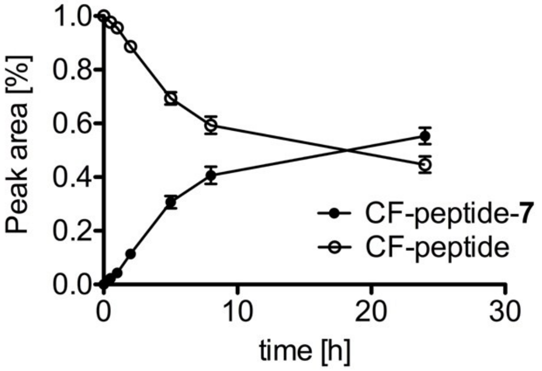

Supplementary Figure 5. Ligation efficiency of ethynyloxy-tyrosine (**7**) to the Tub-tag peptide **3**. UPLC-MS traces were taken at different time points of the TTL reaction and quantitation of substrate and product was performed through peak integration as described before. The mean values and standard deviation (SD) of three replicate reactions are shown.

1.5 Ligation of 10 to peptide 3

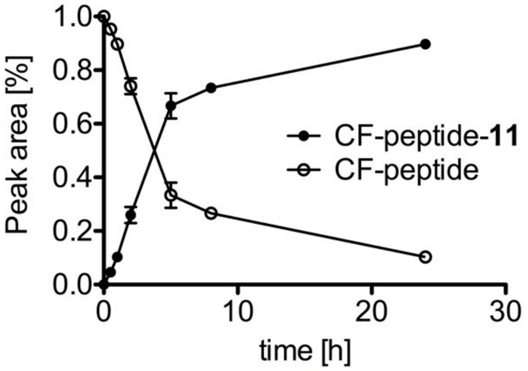

Supplementary Figure 6. Ligation efficiency of tryptophan (**10**) to the Tub-tag peptide **3**. UPLC-MS traces were taken at different time points of the TTL reaction and quantitation of substrate and product was performed through peak integration as described before. The mean values and standard deviation (SD) of three replicate reactions are shown.

1.6 Ligation of 14 to peptide 3

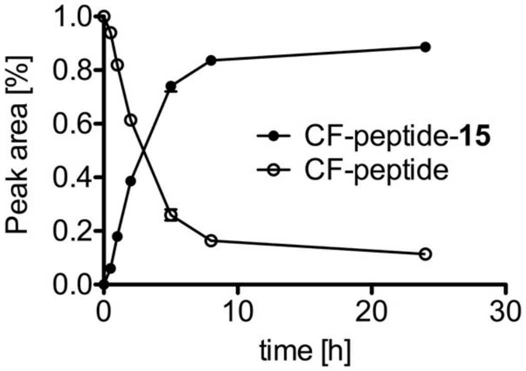

Supplementary Figure 7. Ligation efficiency of 5-F-tryptophan (**14**) to the Tub-tag peptide **3**. UPLC-MS traces were taken at different time points of the TTL reaction and quantitation of substrate and product was performed through peak integration as described before. The mean values and standard deviation (SD) of three replicate reactions are shown.

1.7 Coumarin labeling of Ubiquitin-Tub-tag

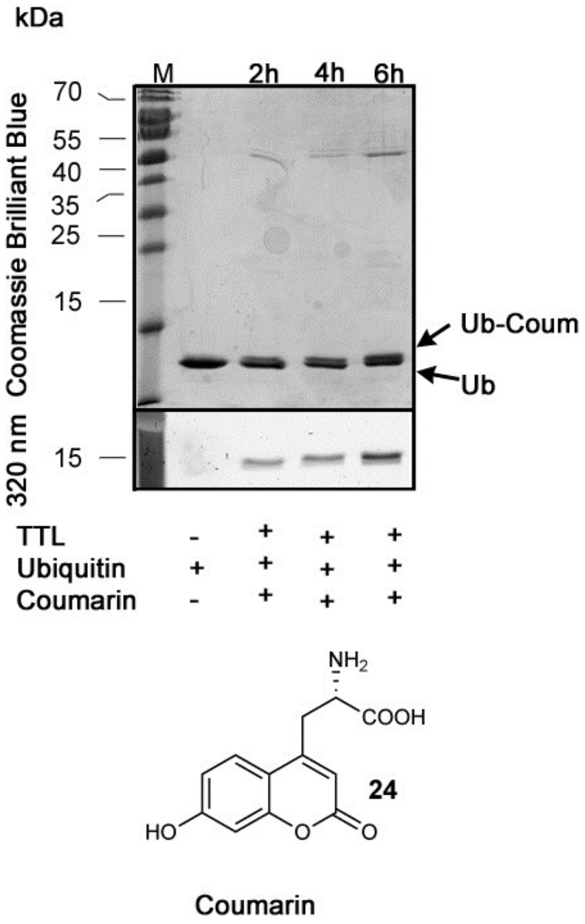

Supplementary Figure 8. SDS-PAGE analysis of the TTL catalyzed coumarin **24** incorporation to ubiquitin. Ub: Ubiquitin, Ub-Coum: Ubiquitin with incorporated coumarin derivative, TTL: tubulin-tyrosine ligase.

1.8 Coumarin labeling of GBP4-Tub-tag

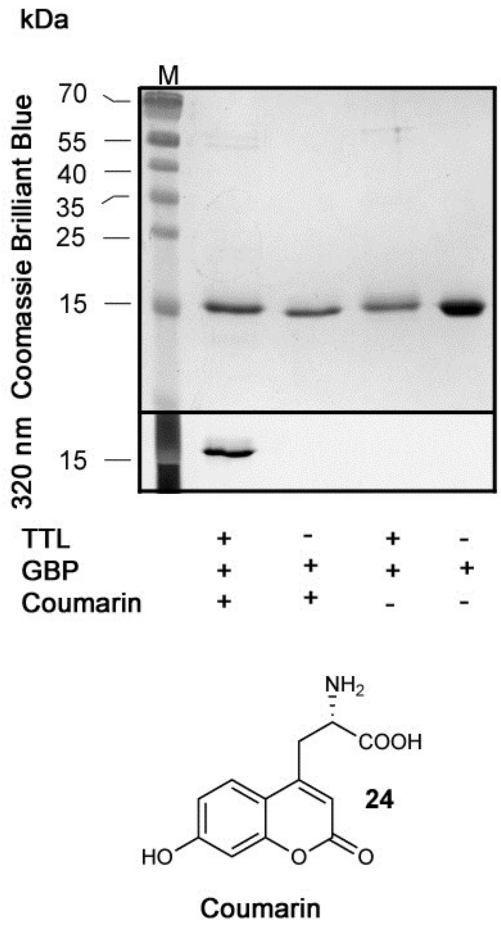

Supplementary Figure 9. SDS-PAGE analysis of the TTL catalyzed coumarin **24** incorporation to a GFP binding nanobody (GBP). TTL: tubulin-tyrosine ligase.

1.9 Immunofluorescence of GFP fusion proteins

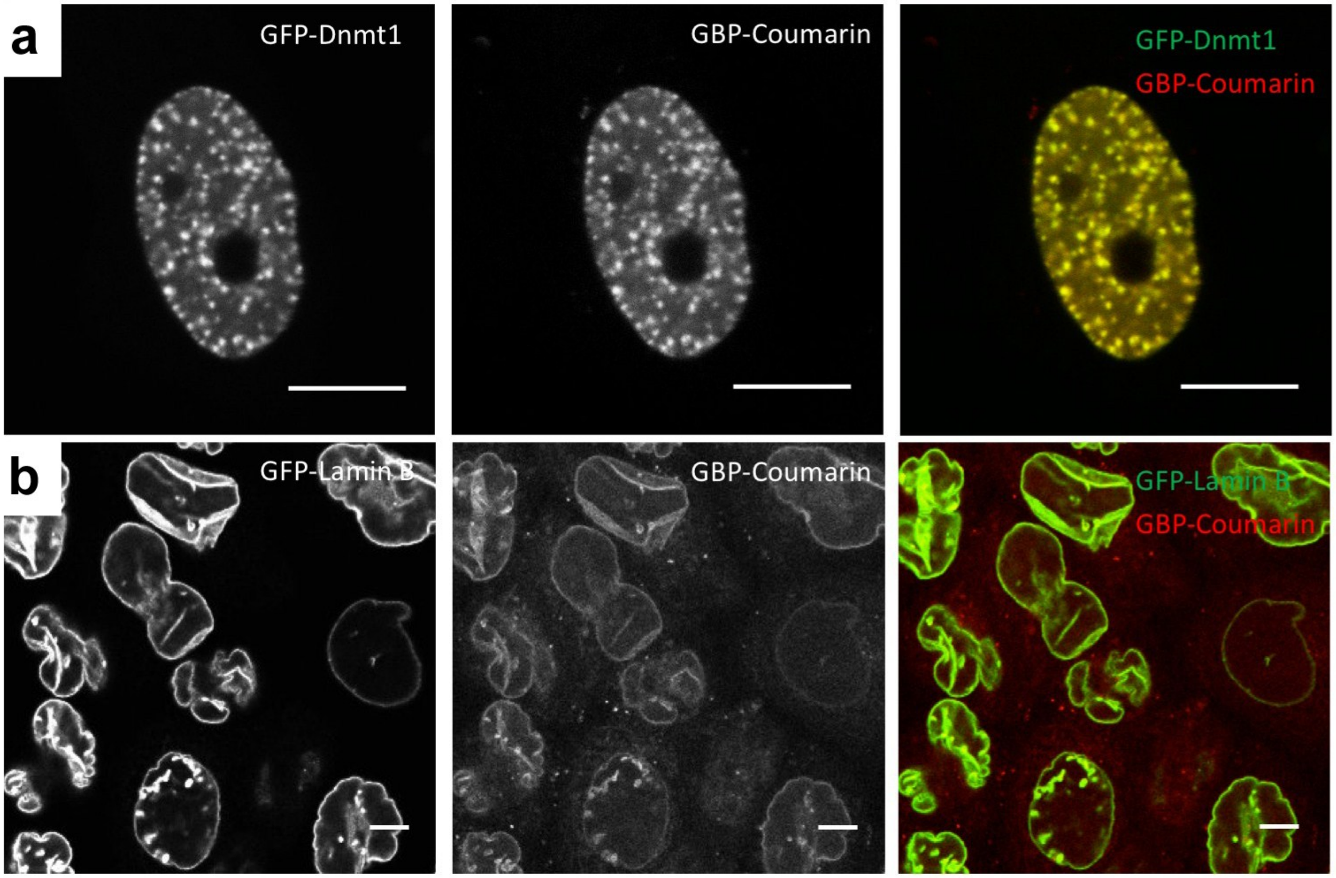

Supplementary Figure 10. Immunofluorescence of GFP-fusion proteins with a GFP-specific nanobody (GBP4), functionalized with coumarin **24** via Tub-tag mediated coumarin incorporation. (a) Co-localization with GFP-Dnmt1. (b) Co-localization with GFP-LaminB1. Scalebar is 5 μm.

1.10 Coumarin labeling of Annexin V-Tub-tag

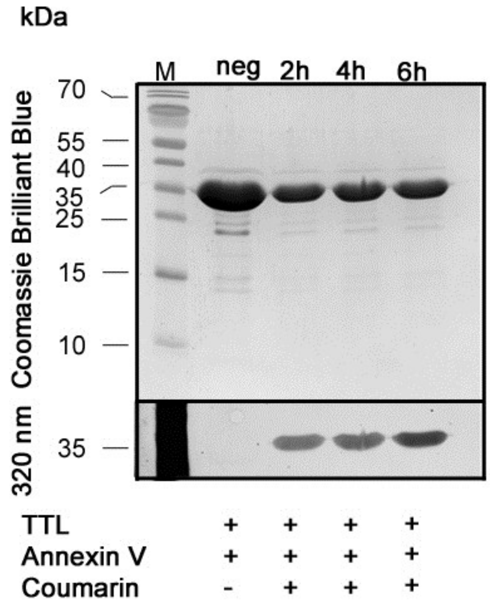

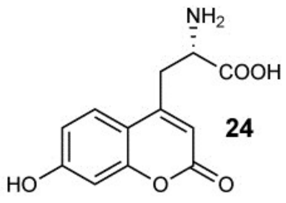

24

Coumarin

Supplementary Figure 11. SDS-PAGE analysis of the TTL catalyzed coumarin **24** incorporation to the apoptosis marker Annexin V. TTL: tubulin-tyrosine ligase.

1.11 Detection of apoptotic cells with Annexin V_Coumarin

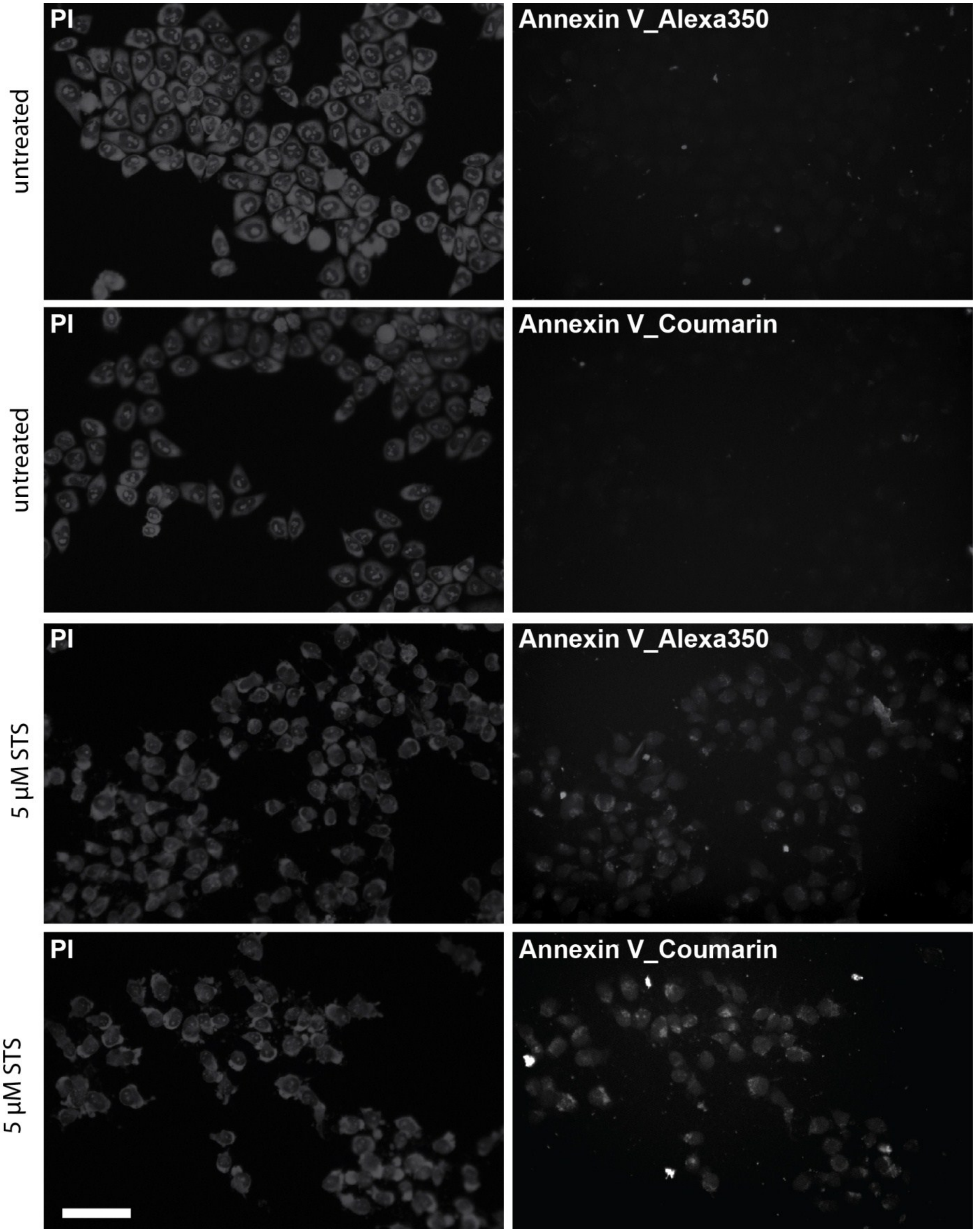

Supplementary Figure 12. Detection of apoptotic cells with Annexin V_Coumarin and commercial Annexin V_Alexa350. Staurosporine-treated (5 µM; lower panels) and untreated cells (upper panels) were stained with commercial Annexin V_Alexa350 or Annexin V_Coumarin, generated via Tub-tag mediated functionalization and counterstained with Propidium Iodide.

2. Material and Methods

2.1 General Information

Analytical HPLC was conducted on a SHIMADZU HPLC system (Shimadzu Corp., Japan) with a SIL-20A autosampler, 2 pumps LC2 AAT, a 2489 UV/Visible detector, a CTO-20A column oven and an RF-10 A X2 fluorescence detector using an Agilent Eclipse C18 5 µm, 250 x 4.6 mm RP-HPLC-column with a flow rate of 0.5 mL/min. The following gradient was used: Method A: (A = H_2O + 0.1% TFA, B = MeCN + 0.1% TFA) 35% B, 0-15 min, 10-100% B 15-17 min, 100% B 17-22 min, 100-35% B 22-25 min and 35% B 25-30 min. UV chromatograms were recorded at 220 nm and fluorescence spectra with Ex/Em 495/517 were recorded.

Analytical UPLC: UPLC-UV traces were obtained on a Waters H-class instrument equipped with a Quaternary Solvent Manager, a Waters autosampler and a Waters TUV detector connected to a 3100 mass or QDa™ detector with an Acquity UPLC-BEH C18 1.7 µm, 2.1 x 50 mm RP column with a flow rate of 0.5 mL/min (Water Corp., USA). The following gradient was used: Method B: (A = H_2O + 0.1% TFA, B = MeCN + 0.1% TFA) 5% B 0.5 min, 5-95% B 0.9-3 min, 95% B 3-5 min. UPLC-UV chromatograms were recorded at 220 nm. Method BII: (A = H_2O + 0.1% TFA, B = MeCN + 0.1% TFA) 5-95% B 0-15 min, 95% B 15-20 min. UPLC-UV chromatograms were recorded at 220 nm.

Preparative HPLC was performed on a Gilson PLC 2020 system (Gilson Inc., WI, Middleton, USA) using a Macherey-Nagel Nucleodur C18 HTec Spum column (Macherey-Nagel GmbH & Co. Kg, Germany). The following gradient was used: Method C: (A = H_2O + 0.1% TFA, B = MeCN + 0.1% TFA) flow rate 32 mL/min, 10% B 0-5 min, 10-100% B 5-35 min, 100 % B 35-40 min. Method D: (A = H_2O + 0.1% TFA, B = MeCN + 0.1% TFA) 10% B 0-5 min, 10-100% B 5-50 min, 100% B 50-55 min.

Analytical HPLC-MSMS: Peptides were analyzed by a Ultimate 3000 nanoLC system (Thermo Scientific, USA) connected to an LTQ Orbitrap XL mass spectrometer (Thermo Scientific, USA). LC separations were performed on a capillary column (Acclaim PepMap100, C18, 3 µm, 100 Å, 75 µm i.d. x 25 cm, Thermo Scientific, USA) at an eluent flow rate of 300 nL/min. The following gradient was used: Method D: (A = H_2O + 0.1% formic acid, B = MeCN + 0.1% formic acid) 3-50% B 0-50 min Mass spectra were acquired in a data-dependent mode with one MS survey scan with a resolution of 30,000 (LTQ Orbitrap XL) or 60,000 (Orbitrap Elite) and MS/MS scans of the five most intense precursor ions in the linear trap quadrupole, respectively.

Column chromatography was performed on silica gel (Acros Silica gel 60 Å, 0.035-0.070 mm).

High-resolution mass spectra (HRMS) were measured on an Acquity UPLC system and a LCT Premier™ (Waters Corp., USA) time-of-flight mass spectrometer with electrospray ionization using water and acetonitrile (10-90% gradient) with 0.1% formic acid as eluent.

NMR spectra were recorded with a Bruker Ultrashield 300 MHz spectrometer (Bruker Corp., USA) at ambient temperature. The chemical shifts are reported in ppm relative to the residual solvent peak. Product yields were calculated based on ^{1}H-NMR spectra. TFA salt content was determined by ^{19}F-NMR, tetrafluoroethylene as standard and considered in product yield calculation.

Reagents and solvents were, unless stated otherwise, commercially available as reagent grade and did not require further purification. Resins and Fmoc-protected amino acids were purchased from IRIS BioTEch (Germany) or Novabiochem (Germany). Tryptophan derivatives **12-17** were purchased from Biosynth AG (Switzerland) and used withouth further purification.

SPPS was either carried out manually or with an Activo-P11 automated peptide synthesizer (Activotec, UK) via standard Fmoc-based conditions (Fast-moc protocol with HOBt/HBUT conditions).

2.2 Experimental Procedures

2.2.1 TTL expression and purification

TTL was expressed and purified according to a published protocol[1]. The TTL (*Canis lupus*) coding sequence was amplified from a mammalian expression vector[2], cloned into a pET28-SUMO3 (EMBL-Heidelberg, Protein Expression Facility) and expressed in *E. coli* BL21(DE3) as Sumo-TTL fusion protein with an N-terminal His-Tag. Cells were induced with 0.5 mM IPTG and incubated at 18 °C for 18 h. Lysis was performed in presence of Lysozyme (100 µg/ml), DNAse (25 µg/ml) and PMSF (2 mM) followed by sonification (Branson® Sonifier; 16 x 8sec, 20% amplitude) and debris centrifugation at 20.000 g for 30 min. His-Sumo-TTL was purified using a 5ml His-Trap. Purified protein

was then desalted on a PD10 column (GE Healthcare); buffer was exchanged to MES/K pH 6.8 (20 mM MES, 100 mM KCl, 10 mM $MgCl_2$). Protein aliquots were shock-frozen and stored at -80 °C at 2.7 g/l.

2.2.2 Determination of TTL substrate scope using CF–Tub-tag peptide

Tyrosination reactions were performed in a 250 µL solution consisting of 20 mM MES/K pH 7.0, 100 mM KCl, 10 mM $MgCl_2$, 2.5 mM ATP, 1 mM amino acid derivative, 0.2 mM CF–Tub-tag, 1 µM TTL and 5 mM DTT. The mixture was incubated at 37 °C and several aliquots (25 µL) were taken, mixed with equal volumes of H_2O + 0.1% TFA and subjected to analytical UPLC (Method B) and isocratic analytical HPLC (Method A). Quantities of substrate and product peptides were estimated from the corresponding peak-area in the TIC, UV or fluorescence detection spectrum (Ex/Em: 495/517).

2.2.3 GBP–Tub-tag expression and purification

The expression and purification of the GBP nanobody was performed according to a previously published protocol[1]. GBP–Tub-tag fusion expression constructs (pHen6 bacterial expression vector) were generated by standard molecular biology techniques resulting in nanobodies with an N-terminal 6xHis tag and a C-terminal Tub-tag. Proteins were expressed in *E. coli* (JM109). Cells were induced with 0.5 mM IPTG and incubated at 18 °C for 18 h. Lysis was performed in presence of Lysozyme (100 µg/ml), DNAse (25 µg/ml) and PMSF (2 mM) followed by sonication (Branson® Sonifier; 16 x 8sec, 20% Amplitude) and debris centrifugation at 20.000 g for 30 min. The protein was purified with an Äkta FPLC system using a 5 mL His-Trap (GE Healthcare, USA) column, peak fractions were concentrated to 2 ml using Amicon filter columns (cut-off 3 kDa; (Merck Millipore, Germany) and subjected to size exclusion chromatography using a Superdex 75 column (GE Healthcare, USA). Peak fractions were pooled and protein aliquots were shock-frozen and stored at -80 °C.

2.2.4 Ubiquitin-Tub-tag expression and purification

The expression and purification of ubiquitin-Tub-tag was performed according to a previously published protocol[1]. Proteins were expressed in *E. coli* BL21 (DE3). Cells were induced with 0.5 mM IPTG and incubated at 37 °C for 5 h. Lysis was performed using a high-pressure homogenizer (Micrufluidics LM10 Microfluidizer) and debris centrifugation at 20.000 g for 30 min. The protein was purified with an NGC™ Chromatography System (BioRad, USA) using a 5 mL His,Trap (GE Healthcare, USA) column, peak fractions were concentrated to 2 mL using Amicon filter columns (cut-off 3 kDa (Merck Millipore, Germany) and futher purified by size-exclusion chromatography (Superdex 75 column, GE Healthcare, USA). Peak fractions were pooled and protein aliquots were shock-frozen and stored at ,80 °C

2.2.5 Annexin V-Tub-tag expression and purification

The coding sequence of Annexin V in fusion with a C-terminal Tub-tag sequence was cloned into pet22b bacterial expression vector using standard molecular biology techniques. Annexin V was expressed in *E. coli* BL21 (DE3). Cells were induced with 1 mM IPTG and incubated at 37 °C for 3 h. Lysis was performed in PBS (1.8 mM KH_2PO_4, 10 mM Na_2HPO_4, 2.7 mM KCl and 137 mM NaCl, pH 7.4) using a high-pressure homogenizer (Micrufluidics LM10 Microfluidizer) and debris centrifugation at 20.000 g for 30 min. The protein was purified with an NGC™ Chromatography System (BioRad, USA) using a 5 mL GST-Column (Bio-Scale™ Mini-Profinity™ GST, BioRad, USA), protein eluted with 500 mM glutathione in PBS and peak fractions desalted and concentrated to 2 mL using Amicon filter columns (cut-off 5 kDa (Merck Millipore, Germany). Precission protease (2000 u/mL, GE Healthcare, USA) was added to the protein fractions and incubated for 16 h at 16°C. To remove the free GST, the solution was applied to another purification using 5 mL Bio-Scale Mini Profinity GST Cartridge (BioRad, USA) as described above. The flowthrough fraction was collected and concentrated to 1 mL using Vivaspin 20 (cut-off 3 kDa; Merck Millipore, Germany) and subjected to a final size exclusion chromatography in PBS using a Superdex 75 10/300 GL column (GE Healthcare, USA). Peak fractions were pooled and aliquots were shock-frozen and stored at -80 °C until further use.

2.2.6 Ligation of coumarin-derivative 24 to ubiquitin

Functionalization reactions were performed in a 150 µL solution consisting of 20 mM MES/K pH 7.0, 100 mM KCl, 10 mM $MgCl_2$, 2.5 mM ATP, 1 mM (**24**), 1 µM TTL, 5 µM ubiquitin and 5 mM DTT. The mixture was incubated at 37° for 1-6 h. Proteins were separated and analysed by SDS-PAGE and a ChemiDoc™ XRS+ gel imaging system (Bio-Rad, Hercules, CA, US).

2.2.7 Ligation of coumarin-derivative 24 to GBP

Functionalization reactions were performed in a 150 µL solution consisting of 20 mM MES/K pH 7.0, 100 mM KCl, 10 mM $MgCl_2$, 2.5 mM ATP, 1 mM (**24**), 1 µM TTL, 5 µM nanobody and 5 mM DTT. The mixture was incubated at 37° for 1-6 h. Proteins were separated and analysed by SDS-PAGE and a ChemiDoc™ XRS+ gel imaging system (Bio-Rad, Hercules, CA, US).

2.2.8 Ligation of coumarin-derivative 24 to Annexin V

Functionalization reactions were performed in a 150 µL solution consisting of 20 mM MES/K pH 7.0, 100 mM KCl, 10 mM $MgCl_2$, 2.5 mM ATP, 1 mM (**24**), 1 µM TTL, 5 µM nanobody and 5 mM DTT. The mixture was incubated at 37° for 1-6 h. Proteins were separated and analysed by SDS-PAGE and a ChemiDoc™ XRS+ gel imaging system (Bio-Rad, Hercules, CA, US).

2.2.9 Immunofluorescence for confocal microscopy

Coumarin-functionalized GFP binding nanobody (GBP-Coumarin) was used for immunostaining. First, HeLa cells were seeded on coverslips in 6-well plates (Greiner, Germany), transfected with plasmids encoding GFP-PCNA[3], GFP-Dnmt1[4] and GFP-LaminB1[5] using lipofectamine transfection reagent (Life Technologies). 24 h post transfection, cells were washed with PBST, cells were fixed in 3.7% formaldehyde in PBS for 10 min, permeabilized with 0.5% Triton X,100 (neoLab Laborbedarf, Germany) for 10 min, and blocked in 2% bovine serum albumin (Sigma-Aldrich, UK) for 60 min. To stain GFP-fusion proteins, cells were incubated for 60 min with GBP4-Coumarin (1:50 at 1µg/µl) prior to extensive washing and DNA counterstain with 1 µg/mL Propidium Iodide for 10 min. All steps except fixation were carried out in PBS supplemented with 0.02% Tween 20 (PBST, Carl Roth, 9127.1) at room temperature. Glass coverslips were then mounted with vectashield antifade mounting medium (Vector Laboratories,USA).

2.2.10 Annexin V staining

~ $2x10^4$ cells/well were seeded in a 96-well µclear plate (Greiner, Austria). Following 3 h induction of apoptosis with 5 µM Staurosporine (Sigma-Aldrich, UK), cells were stained with either commercial AnnexinV_Alexa350 (5µg/$1x10^5$ cells) or Coumarin-functionalized Annexin V (5µg/$1x10^5$ cells). Cells were fixed in 3.7% formaldehyde in PBS for 10 min at RT, washed with PBS-T and permeabilized with 0.5% Triton X-100 (neoLab Laborbedarf, Germany) for 10 min. Cells were counterstained with Propidium Iodide at 100 µg/ml (Sigma-Aldrich, UK) for 10 min in the dark and followed by repeated washing with PBS-T.

.

2.2.11 Microscopy

Confocal Imaging was carried out with a Leica SP5 II confocal point scanner (Leica Microsystems, Germany). Image acquisition was performed with a 60x/1.4,0.6 NA Planapo-chromat oil immersion objective lens. To visualize Coumarin and GFP the 405 and 488 nm excitation lasers were used, respectively. Microscopic analysis of apoptotic cells, visualized by Annexin V was performed with an Operetta high-content imaging platform (Perkin Elmer, USA). Propidium iodide and Coumarin/Alexa350 were detected using the preset DsRed and DAPI filter combinations.

2.2.12 Docking studies

Docking studies were performed using *AutoDockTools* (*Autogrid4* and *Autodock4*)[6]. For the protein structure the crystal structure with the PDB-ID 4I55[7], which was reduced to TTL, ACP, a part of the Tub-tag (Glu-449 and Glu-450) and Mg-ions was used. Docking was perfomed on a grid of 100 grid points per direction with a spacing of 0.319 Å using a Lamarckian Genetic Algorithm which predicts the free energy of dissociation[8].

2.2.13 Molecular dynamics simulations

All-atom molecular dynamics simulations were performed for ligand-protein-complexes of tyrosine (**4**), tryptophan (**11**) and the coumarin-derivative **24** using explicit solvent in an NVT ensemble. The starting structure for the protein-ligand-complex was imported directly out of *AutoDockTools*. For the simulation, the *GROMACS* simulation package 5.0.2[9], the AMBER ff99SB-ILDN force field[10] as well as TIP3P water model[11] were applied. The parametrization of the ligand was done using *AmberTools* 16[12] as well as *ACPYPE*.[13]

The protein-ligand complex was first energy minimized in vacuum, solved in a dodecahedral box and then energy minimized in solvent. After a 50 ps NVT-equilibration, followed by a 50 ps NPT equilibration, both with position-

restrained protein-ligand complex, a 10 ns NVT simulation, using a position restrain on the protein, was performed. The simulations were carried out using a leap-frog-integrator with a time step of 2 fs at a temperature of 300 K, restrained by the v-rescale thermostat[14] (tau_t = 0.01). The bonds of covalently bond hydrogens were constrained using the LINCS-algorithm[15] (lincs_iter = 1, lincs_order =4). Electrostatic interactions were calculated by a Particle-Mesh Ewald summation[16] using a real space cutoff of 1 nm, a Fourier grid spacing of 0.15 at an interpolation order of 4. The Lennard-Jones interactions were cut off at 1 nm. Solute coordinates of the protein-ligand complex were saved every 1 ps.

2.3. Chemical Synthesis

2.3.1 Synthesis of 3-formyl-*L*-tyrosine (S3)

The synthesis of S3 was performed according to a known procedure in literature[1, 17, 18].

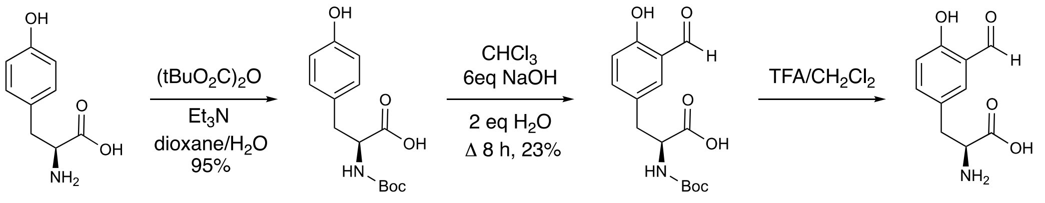

S14 **S15** **S16**

Supplementary Scheme 1. Synthesis of 3-formyl-*L*-tyrosine (S3).

N-[(1,1-dimethylethoxy)carbonyl]-*L*-tyrosine (**S14**)

To a solution of *L*-tyrosine (1 g, 5.5 mmol) in 1/1 dioxane/water (50 mL), triethylamine (1.16 mL, 8.28 mmol) was slowly added. The reaction was cooled to 0°C with an ice/water bath and *di*-*tert*-butyl dicarbonate (1.32 g, 6.07 mmol) was added in two steps. After 1 h at 0°C, the temperature was slowly increased to ambient temperature and the mixture was stirred for further 24 h. Dioxane was removed under reduced pressure and the aqueous solution mixed with saturated $NaHCO_3$ (25 mL), washed with ethyl acetate, acidified to pH 1 with 1 N HCl, extracted with ethyl acetate and the organic extracts were washed with brine, dried over $MgSO_4$ and evaporated to give Boc protected tyrosine **S14** as a white foam (1.471 g, 95%) which was used in the next step without further purification. Analytical data matched the literature[17].

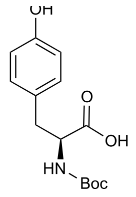

S14

^{1}H-NMR (300 MHz, $CDCl_3$): δ 7.50-7.22 (m, 2H, CH_{phenyl}), 7.42 (dd, *J* = 8.6, 2.3 Hz, 1H, CH_{phenyl}), 6.92 (d, *J* = 8.4 Hz, 1H, CH_{phenyl}), 5.11 (br, 1H, N*H*), 4.73-4.28 (m, 1H, C*H*), 3.32-2.90 (m, 2H,CH_2), 1.42 (s, 9H, CH_3).

N-[(1,1-dimethylethoxy)carbonyl]-3-(3-formyl-4-hydroxyphenyl)-*L*-alanine (**S15**)

To a suspension of **S14** (2.00 g, 7.12 mmol) in chloroform (30 mL) and H_2O (0.256 mL, 14.13 mmol) powdered sodium hydroxide (1.71 g, 42.72 mmol) was added and the mixture was refluxed for 4 h. Two additional portions of powdered sodium hydroxide (each 0.42 g, 10.68 mmol) were added after 1 and 2 h. After 8 h at reflux, the reaction was cooled to ambient temperature, diluted with water and ethyl acetate (15 mL each), the organic layer discharged, the aqueous layer acidified to pH 1 with 1 N HCl and back-extracted with ethyl acetate. The organic layers were

washed with brine, dried over $MgSO_4$ and concentrated. Flash column chromatography (silica gel, 12/1 $CHCl_3$/MeOH, 1% acetic acid) gave compound **S15** (0.49 g, 23%). Analytical data matched the literature[17].

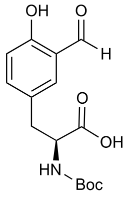

S15

^{1}H-NMR (300 MHz, $CDCl_3$): δ 9.85 (s, 1H, C*H*O), 7.49-7.21 (m, 2H, CH_{phenyl}), 7.40 (dd, *J* = 8.6, 2.3 Hz, 1H, CH_{phenyl}), 6.94 (d, *J* = 8.4 Hz, 1H, CH_{phenyl}), 5.10 (br, 1H, N*H*), 4.73-4.27 (m, 1H, C*H*), 3.30-2.89 (m, 2H,CH_2), 1.40 (s, 9H, CH_3).

3-formyl-*L*-tyrosine (**S16**)

Compound **S15** (0.49 g, 1.6 mmol) was dissolved in CH_2Cl_2. TFA (4 mL) was added slowly at 0°C and the mixture was warmed to ambient temperature within 2 h. The solvent was removed at high vacuum. Preparative HPLC (method C) gave compound **S16** as TFA salt (0.29 g, 80%). Analytical data matched the literature[17].

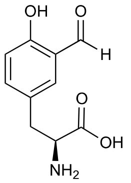

S16

^{1}H-NMR (300 MHz, D_2O): δ 9.81 (s, 1H, CHO), 7.52 (d, *J* = 2.4 Hz, 1H, CH_{phenyl}), 7.40 (dd, *J* = 8.6, 2.3 Hz, 1H, CH_{phenyl}), 6.90 (d, *J* = 8.6 Hz, 1H, CH_{phenyl}), 4.13 (t, *J* = 6.6 Hz, 1H, CH), 3.15 (m, 2H,CH_2); ^{13}C-NMR (75 MHz, D_2O): δ 197.18, 171.68, 159.21, 138.07, 134.02, 126.03, 120.97, 117.73, 54.18, 34.48; ESI-HRMS (*m/z*):[M]$^+$ calcd. for $C_{10}H_{12}NO_4$, 210.0758; found 210.0760.

2.3.2 Synthesis of Tyr(*o*-propargyl) (7)

The synthesis of the Tyr(*o*-propargyl) was performed according to a known procedure in literature[19].

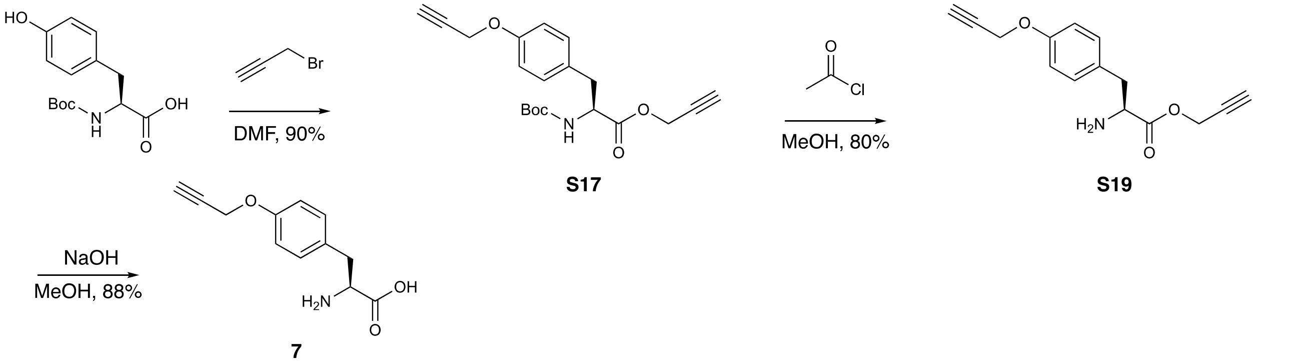

Supplementary Scheme 2. Synthesis of compound 7.

Intermediate **S17**

Boc-L-Tyr-OH (2.51 g, 8.9 mmol) and K2CO3 were suspended in dry DMF (20 mL). Propargyl bromide (80% in toluene, 2.88 mL, 26.75 mmol) was slowly added stirred at ambient temperature for 24h. H2O and Et2O (50 mL each) were added and the org. phase separated and the aqueous layer extracted with Et2O (2x 40 mL). The combined org. phases were dried over MgSO4 and evaporated under reduced pressure to give 2.85 g (90%) of intermediate **S17**. The compound was used without further purification.

Intermediate **S5**

Acetyl chloride (7.27 g, 658 mL, 92.6 mmol) was slowly added to dry methanol (55 mL) at 0°C and added to compound **S17** (6.02 g, 16.86 mmol) at 0°C and slowly warmed to ambient temperature. The mixture was stirred for additional 20h and all volatile compounds removed to give the HCl salt of **S18** as a white solid (4.01 g, 13.63 mmol, 80%). The compound was used without further purification.

Tyr(o-propargyl) (**7**)

S18 (4.01 g, 13.63 mmol) was dissolved in MeOH (15 mL) and aqueous 2N NaOH (20 mL) was added slowly. The mixture was stirred at ambient temperature for 20 h and acidified with conc. HCl and stored at 4°C for 20 h. The white precipitate was filtered of and dried in the vacuum to yield the HCl-salt of **7** (3.05 g, 11 mmol, 88%). Analytical data matched the literature[19].

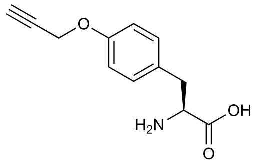

7

^{1}H NMR (300 MHz, DMSO-d_6) δ 7.20 (d, J = 8.3 Hz, 2H), 6.90 (d, J = 8.3 Hz, 2H), 4.75 (d, J = 2.4 Hz, 2H), 3.56 (t, J = 2.3 1H), 3.45 (dd, J = 7.8, 4.6 Hz, 1H), 3.08 (dd, J = 14.4, 4.6 Hz, 1H), 2.85 (dd, J = 14.4, 7.9 Hz, 1H). ^{13}C-NMR (151 MHz, DMSO-d_6) δ, 170.18, 156.46, 130.89, 130.38, 115.11, 79.91, 78.56, 55.87, 55.78, 40.50, 36.35, 34.56; ESI-MS (m/z):[M]$^+$ calcd. for $C_{12}H_{13}NO_3$, 219.09; found 219.09

2.3.3 Synthesis of (1S)-1-Carboxy-2-(7-hydroxy-2-oxo-2H-chromen-4-yl)ethyl ammonium trifluoroacetate (24)

The synthesis of coumarin-derivative **24** was performed according to a known procedure in literature[20].

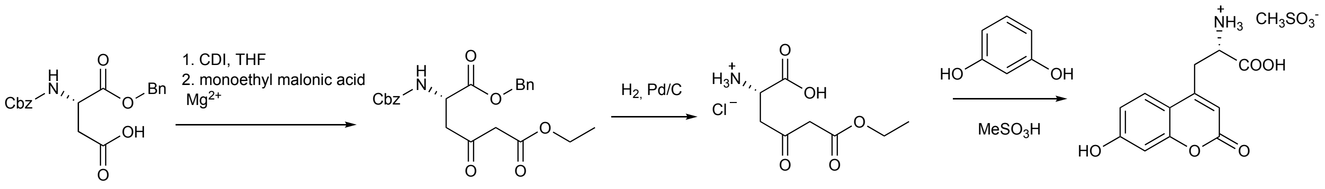

Supplementary Scheme 3. Synthesis of coumarin-derivative 24

(2S)-2-Benzyloxycarbonylamino-4-oxo-hexanedioic acid 1-benzyl ester 6-ethyl ester (**S19**)

A solution of Cbz-(L)-Asp-OBn and carbonyldiimidazole (1.1 eq) in THF was stirred for two hours at ambient temperature, cooled to 0°C and 0.54 eq. of monoethyl malonic acid magnesium salt was added. The mixture was stirred overnight at ambient temperature. The mixture was diluted with ether (15 mL) and acidified with conc. HCl at 0°C and the two phases separated. The organic phase was washed with 10% $NaHCO_3$, 4:1 H_20:$KHSO_4$(1M), H_20 and brine, dried over MgSO4 and the solvent removed. A final flash purification (1:1 EtOAc:hexane) resulted in 1.67 g (70 %) of compound **S19** as a white solid. The analytical data matched the literature[20].

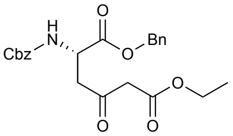

S19

1H NMR (300 MHz, CDCl3) δ 7.38 – 7.28 (m, 12H), 5.98 (d, J = 8.5 Hz, 1H), 5.16 (s, 2H), 5.11 (s, 2H), 4.73 – 4.61 (m, 1H), 4.15 (q, J = 7.2 Hz, 2H), 3.27 (dd, J = 18.5, 4.9 Hz, 1H), 3.13 (dd, J = 18.4, 4.4 Hz, 1H), 1.23 (t, J = 7.2 Hz, 3H). ^{13}C-NMR (75 MHz, DMSO-d_6): 200.72, 161.51, 155.40, 135.71, 134.83, 128.31, 128.24, 128.01, 127.84. 127.73, 67.27, 61.21, 49.53, 48.74, 44.35

(1S)-1-Carboxy-2-(7-hydroxy-2-oxo-2H-chromen-4-yl)ethyl ammonium trifluoroacetate (**24**)

Compound **S19** was dissolved in 10 mL of 1:1 AcOEt:95% EtOH and 1 eq. 1N HCl and 0.05 eq. of10% Pd on charcoal was added and stirred for 2h at ambient temperature. The Pd was filtered off, washed with 95% EtOH and the filtrate concentrated. The residue was taken up in water and lyophilised to give 0.429 g (90 %) of compound **S7**. The compound was used without further purification. Compound **S20** (200 mg, 0.98 mmol) and 3-hydroxyphenol (0.161 g, 1.47 mmol) were mixed and 99% methansulfonic acid (1.59 mL, 25 eq.) added at 0°C and stirred at ambient temperature for two additional hours. The mixture was taken up in cold ether and centrifuged 20 minutes at 4000g, the ether removed and the residue taken up in water. A final preparative HPLC (Method C) purification gave 0.136 g (40 %) of Coumarin derivative **24**. Fluorescence emission and absorption spectra were measured.

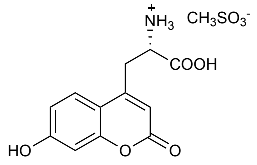

24

^{1}H-NMR (300 MHz, DMSO-d_6): δ 10.72 (s, 1H), 8.57 – 7.88 (br, 3H), 7.61 (d, *J* = 8.7 Hz, 1H), 6.84 (dd, *J* = 8.8, 2.4 Hz, 1H), 6.76 (d, *J* = 2.3 Hz, 1H). 6.20 (s, 1H). 4.08 (dd, *J* = 9.2, 5.0 Hz, 1H), 3.39 (dd, *J* = 10.2 Hz, 1H), 3.05 (dd, *J* = 14.6, 9.3 Hz, 1H). ^{13}C-NMR (75 MHz, DMSO-d_6): 169.97, 161.77, 160.49, 155.77, 150.92, 126.50, 113.50, 112.89, 111.29. 103.05, 52.11, 32.97; ESI-MS (*m/z*):[M]$^{+}$ calcd. for $C_{12}H_{13}NO_5$, 250.07; found 250.07.

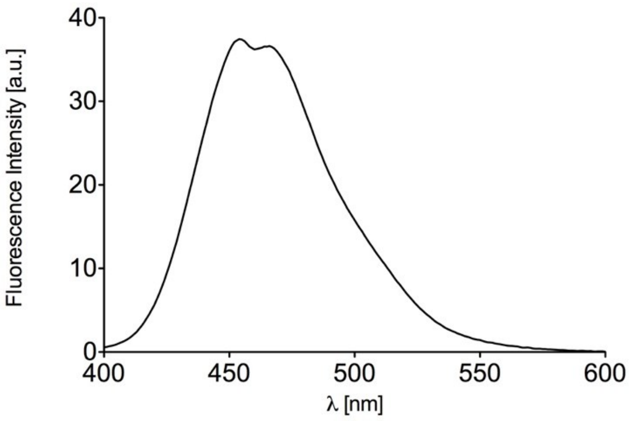

Supplementary Figure 13. Emission spectrum of derivative **24**

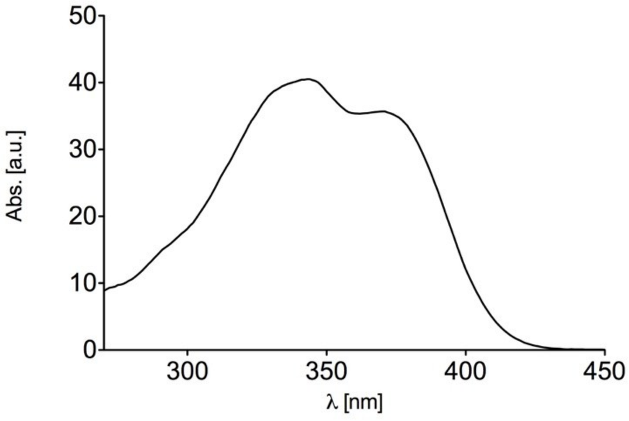

Supplementary Figure 14. Absorption spectrum of derivative **24**

2.3.4 Synthesis of Azuelenyl-alanine 25

Azulene (**S21**)

To mechanically stirred anhydrous pyridine (400 mL) under an N_2 atmosphere was added 1-chloro-2,4-dinitrobenzne (101.27 g, 500 mmol). The reaction mixture was then heated at 85 °C for 3 h, after which a thick yellow precipitate formed. The slurry was then cooled to 0 °C and a solution of dimethylamine (68.01 g, 1.50 mol) in pyridine (200 mL) at 0 °C was added over 15 min. The reaction mixture was then allowed to warm to room temperature and stirred overnight. After this time, freshly cracked cyclopentadiene (35.0 g, 530 mmol) was added NaOMe (27.1 g, 500 mmol) in MeOH (200 mL) and stirred for 4 h. The mixture of methanol and pyridine was distilled out of the reaction mixture until the vapour temperature reached 110 °C. The reaction mixture was then allowed to cool, and pyridine (300 mL) was added and heated under reflux for 4 days, after which the temperature was lowered to 60 °C and the pyridine was removed under reduced pressure. The resultant black residue was then placed into a Soxhlet apparatus and extracted with hexane for 3 days. The resultant dark green solution was then distilled through a Vigreux column (taking care to ensure no blue colour is present in the distillate). The remaining blue concentrate was purified by alumina flash chromatography eluting with hexane to afford the product (7.25 g, 11%) as a blue solid. Analytical data were consistent with those previously reported.[21]

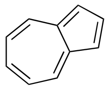

S21

R_f 0.95 (MTBE); m.p. 98-98.5 °C; lit m.p. 99 °C; ^{1}H NMR (400 MHz, $CDCl_3$) δ 8.38 (2H, d, *J*=10.0 Hz, **4**-H & **8**-H), 7.94 (1H, t, *J*=4.0 Hz, **2**-H), 7.62 (1H, t, *J*=10.0 Hz, 1-H, **3**-H), 7.43 (2H, d, *J*=4.0 Hz, **1**-H & **3**-H), 7.20 (2H, t, *J*=10.0 Hz, **5**-H & **7**-H); ^{13}C NMR (101 MHz, $CDCl_3$) δ 140.1 (**3a**-C & **8a**-C), 137.2 (**6**-C), 137.0 (**2**-C), 136.6 (**4**-C & **8**-C), 122.8 (**5**-C & **7**-C), 118.0 (**1**-C & **3**-C); *m/z* APCI$^+$ 87.08 (unknown, 100%), 129.07 ([M+H]$^+$, 50%).

1-(Azulen-1-yl)-*N,N*-dimethylmethanamine (**S22**)

A mixture of paraformaldehyde (163 mg, 5.45 mmol), *N,N'*-tetramethyldiaminomethane (614 mg, 6.00 mmol) and glacial acetic acid (17 mL) was heated until the solution became transparent. The solution was then cooled and added to a solution of azulene (1.39 g, 10.92 mmol) in CH_2Cl_2 (30 mL) at 0 °C and stirred for 3 h. The reaction mixture was then treated with 1 M HCl (100 mL) and extracted with CH_2Cl_2 (3 × 50 mL). The aqueous layer was then basified to pH 14 with 1 M KOH, and then extracted with MTBE (6 × 50 mL). The combined organic washings were dried (Na_2SO_4) and concentrated *in vacuo* to afford the product (1.31 g, 65%) as a dark blue oil. Analytical data were consistent with those previously reported.[22]

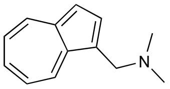

S22

R_f 0.00 (EtOAc); ^{1}H NMR (400 MHz, $CDCl_3$) δ 8.50 (1H, d, *J*=9.5 Hz), 8.31 (1H, d, *J*=9.5 Hz), 7.89 (1H, d, *J*=4.0 Hz), 7.59 (1H, t, *J*=10.0 Hz), 7.36 (1H, d, *J*=4.0 Hz), 7.18 (1H, dd, *J*=10.0, 9.5 Hz), 7.14 (1H, dd, *J*=10.0, 9.5 Hz), 3.92 (2H, s), 2.28 (6H, s); ^{13}C NMR (101 MHz, $CDCl_3$) δ 141.1, 138.6, 137.4, 137.1, 136.5, 133.8, 126.9, 122.7, 122.3, 116.6, 56.6, 45.5;

1-(Azulen-1-yl)-*N,N*-dimethylmethanaminium Iodide (**S23**)

To a solution of 1-(Azulen-1-yl)-*N,N*-dimethylmethanamine (1.31 g, 7.05 mmol) in EtOH (50 mL) was added MeI (1.10 g, 7.76 mmol) and stirred for 5 minutes. After this time the reaction mixture was stored overnight at 4°C. The resultant precipitate was filtered and dried *in vacuo* to afford the product (2.20 g, 95%) as a violet solid. Analytical data were consistent with those previously reported.[22]

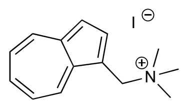

S23

R_f 0.00 (MTBE); ^{1}H NMR (400 MHz, $CDCl_3$) δ 9.15 (1H, d, *J*=9.5 Hz), 8.38 (1H, d, *J*=9.5 Hz), 8.04 (1H, m), 7.75 (1H, t, *J*=9.5 Hz), 7.52 (1H, t, *J*=9.5 Hz), 7.26-7.41 (2H, m), 5.45 (2H, s), 3.39 (9H, s); ^{13}C NMR (101 MHz, $CDCl_3$) δ 142.3, 140.6, 140.0, 139.1, 138.1, 135.7, 126.0, 125.6, 117.8, 113.3, 62.8, 52.5.

Diethyl 2-acetamido-2-(azulen-1-ylmethyl)malonate (**S24**)

To a solution of NaOEt (prepared from Na (1.55 g, 67.59 mmol) in EtOH (200 mL)) was added diethylacetaminomalonate (15.46g, 71.15 mmol) and stirred for 15 minutes. Then 1-(azulen-1-yl)-*N,N*-diemthylmethanaminium iodide (11.64 g, 35.57 mmol) was added and heated under reflux for 2 hours. After this time, the reaction mixture was diluted with H_2O (500 mL) and adjust to pH 7 (1 M HCl) and extracted with MTBE (3 × 200 mL). The organic washings were then dried (Na_2SO_4) and concentrated *in vacuo* to afford the product (10.58 g, 83%) as a dark blue solid. Analytical data were consistent with those previously reported.[22]

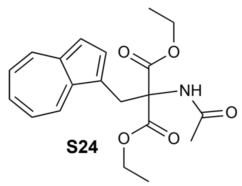

S24

R_f 0.50 CH_2Cl_2:EtOAc (9:1); ^{1}H NMR (400 MHz, $CDCl_3$) δ 8.28 (1H, d, *J*=9.5 Hz), 8.24 (1H, d, *J*=10.0 Hz), 7.53-7.61 (2H, m), 7.32 (1H, d, *J*=4.0 Hz), 7.14 (1H, dd, *J*=9.5, 5.0 Hz), 7.12 (1H, dd, *J*=10.0, 5.0 Hz), 6.48 (1H, br. s), 4.19-4.35 (4H, m), 4.16 (2H, s), 1.95 (3H, s, NAc), 1.32 (6H, t, *J*=7.0 Hz, CH_3); ^{13}C NMR (101 MHz, $CDCl_3$) δ 169.2, 167.7, 140.9, 137.8, 137.7, 137.6, 136.7, 133.6, 122.9, 122.8, 122.1, 117.0, 67.7, 62.6, 30.0, 23.1, 14.0; *m/z* APCI$^+$ 358.16 ($[M+H]^+$, 100%).

2-Acetamido-2-(azulen-1-ylmethyl)malonic acid (**S25**)

A solution of diethyl 2-acetamido-2-(azulen-1-ylmethyl)malonate (4.87 g, 13.63 mmol) in EtOH (100 mL) was mixed with 20% $KOH_{(aq)}$ solution (100 mL) and then heated a reflux for 3 h. The reaction mixture was then cooled to room temperature and diluted with 600 mL H_2O, acidified with 6 M HCl and extracted with MTBE (4 x 400 mL). The combined organic washings were dried (Na_2SO_4) and concentrated *in vacuo* to afford the product (4.10 g, quantitative) as a light blue solid. Analytical data were consistent with those previously reported.[22]

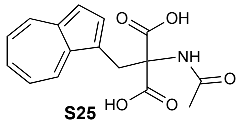

S25

^{1}H NMR (400 MHz, DMSO-d_6) δ 8.22 (d, J=9.9 Hz, 1H), 8.16 (d, *J*=9.4 Hz, 1H), 7.62 (d, *J*=3.5 Hz, 1H), 7.48 (dd, *J*=10.0, 9.5 Hz, 1H), 7.21 (d, *J*=3.5 Hz, 1H), 7.03 (t, *J*=10.0 Hz, 1H), 7.03 (t, *J*=9.5 Hz, 1H), 6.68 (s, 1H), 4.04 (s, 2H), 1.85 (s, 3H); ^{13}C NMR (101 MHz, $CDCl_3$) δ 169.1, 168.7, 140.1, 137.5, 137.1, 136.9, 135.7, 133.1, 123.3, 121.9, 121.4, 116.3, 66.7, 29.1, 22.5.

2-Acetamido-3-(azulen-1-yl)propanoic acid (**S26**)

A solution of 2-acetamido-2-(azulen-1-ylmethyl)malonic acid (4.10 g, 13.62 mmol) in THF (50 mL) was treated with 0.2 M $HCl_{(aq)}$ (275 mL, 54.8 mmol) and heated to 85°C for 8 h. The mixture was then cooled to room temperature and extracted with MTBE (3 x 200 mL). The combined organic extracts were washed with brine, then dried (Na_2SO_4) and concentrated *in vacuo* to afford the product (4.10 g, quantitative) as a dark blue solid.

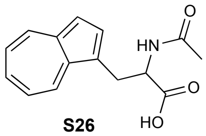

S26

^{1}H NMR (400 MHz, $CDCl_3$) δ 8.19 (d, J=9.5 Hz, 1 H), 8.13 (d, J=9.5 Hz, 1 H), 7.65 (d, J=4.0 Hz, 1 H), 7.44 (t, J=10.0 Hz, 1 H), 7.19 (d, J=4.0 Hz, 1 H), 7.01 (dd, J=10.0, 9.5 Hz, 2 H), 6.98 (dd, J=10.0, 9.5 Hz, 2 H), 6.30 (d, J=7.5 Hz, 1 H), 4.78 (dt, J=7.5, 5.5 Hz, 1 H), 3.59 (dd, J=14.5, 5.5 Hz, 1 H), 3.49 (dd, J=14.5, 5.5 Hz, 1 H), 1.80 (s, 3 H); ^{13}C NMR (101 MHz, $CDCl_3$) δ 173.2, 169.5, 140.4, 137.6, 137.3 (s), 136.9 (s), 136.1 (s), 133.3 (s), 124.2 (s), 122.2 (s), 121.7 (s), 116.7 (s), 53.3 (s), 29.0 (s), 22.8 (s).

(*S*)-2-Amino-3-(azulen-1-yl)propanoic acid (**25**)

A solution of 2-acetamido-3-(azulen-1-yl)propanoic acid (3.61 g, 13.99 mmol) in 4M $KOH_{(aq)}$ (3.5 mL, 14.00 mmol) was added to 0.1 M pH 7.4 Sørensen's phosphate buffer (250 mL) and stirred for 10 minutes. Acylase I from *Aspergillus melleus* (1.2 g) was added to the solution and the mixture was stirred at 37°C overnight. After this time, the mixture was acidified to pH 2.5 with 1M HCl and then filtered through celite. The aqueous solution was then washed with MTBE (2 x 100 mL) to remove the unreacted and undesired enantiomer of the starting material. The aqueous solution was then neutralized to pH 7 using 1M $NaOH_{(aq)}$ solution and concentrated *in vacuo* to a final volume of 50 mL. This deep blue solution was then allowed to stand overnight at 4°C. The resultant precipitate was then filtered and dried *in vacuo* to afford the desired product (1.38, 92% of theoretical resolution maximum) as a dark blue powder. Analytical data were consistent with those previously reported.[22]

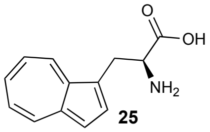

25

^{1}H NMR (500 MHz, D_2O) δ 8.40 (d, *J*=10.0 Hz, 2 H), 7.84 (d, *J*=3.5 Hz, 1H), 7.70 (t, *J*=9.5 Hz, 1H), 7.41 (d, *J*=3.5 Hz, 1H), 7.27 (dd, *J*=10.0, 9.5 Hz, 1 H), 7.23 (dd, *J*=10.0, 9.5 Hz, 1H), 4.05 (dd, *J*=7.5, 5.5 Hz, 1H), 3.72 (dd, *J*=15.0, 5.5 Hz, 1H), 3.56 (dd, *J*=15.0, 7.5 Hz, 1H); ^{13}C NMR (126 MHz, D_2O) δ 174.3, 140.7, 138.6, 137.6, 137.4, 136.6, 133.8, 123.6, 123.0, 121.9, 117.0, 56.1, 28.1.

2.3.4 Synthesis of tyrosine-biotin 26

Supplementary Scheme 4. Synthesis of tyrosine-biotin 26.

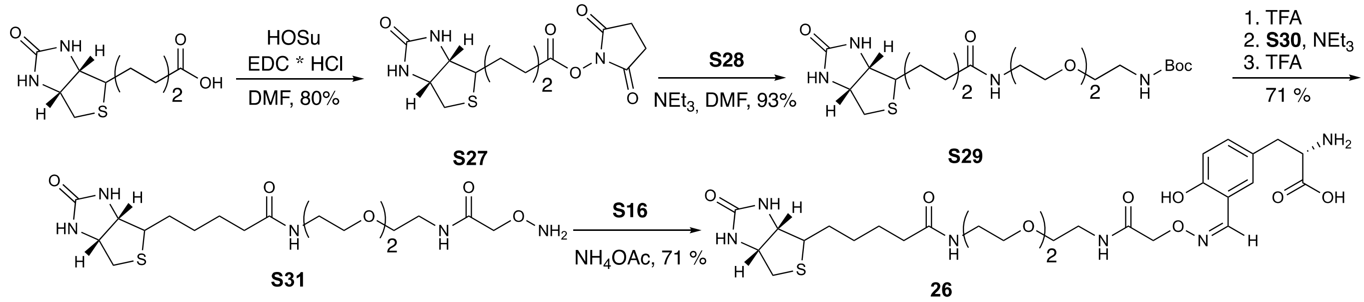

D-biotin *N*-hydroxysuccinimide ester (**S27**)

1-ethyl-3-(3-dimethylaminopropyl)carbodiimid (184 mg, 0.96 mmol) was added to a solution of D-biotin (200 mg, 0.82 mmol) and *N*-hydroxysuccinimid (102 mg, 0.89 mmol) in dry DMF (10 mL). The solution was stirred for 12 h at ambient temperature, concentrated and the product crystallized from 2-propanol to give succinimide ester **S27** (261mg, 80). The product was used without further purification and analytical data are in accordance with those reported in the literature[23].

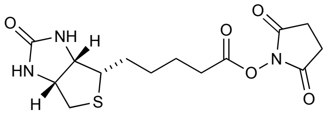

S27

^{1}H-NMR (300 MHz, DMSO): δ 6.41 (s, 1H, N*H*), 6.36 (s, 1H, N*H*), 4.35-4.26 (m, 1H, C*H*), 4.18-4.11 (m, 1H, C*H*), 3.14-3.05 (m, 1H, C*H*), 2.89-2.75 (m, 5H, 2xCH_2, C*H*), 2.64 (t *J* = 7.4 Hz, 2H, CH_2), 2.60-2.55 (m, 1H, 3.32, CH), 1.72-1.32 (m, 6H, 3xCH_2).

2-Amino-3'[(tert-butoxycarbonyl)amino]ethylene glycol diethyl ether (**S28**)

To a solution of 2,2'-(ethylenedioxy)-bis(ethylamine) (4.07 g, 27.46 mmol) and N,N-Diisopropylethylamine (1.56 mL, 9.17 mmol) in dry CH_2Cl_2 (50 mL) at ambient temperature a solution of di-*tert*-butyl dicarbonate (2.0 g, 9.16 mmol) in dry CH_2Cl_2 (20 mL) was added dropwise within 20 min. After additional stirring for 1 h at ambient temperature, the mixture was concentrated, redissolved in 20 mL water and extracted four times with CH_2Cl_2 (10 mL). The organic layers were combined, washed three times with brine, dried with $MgSO_4$ and concentrated to give 2.02 g of a colorless oil of **S28** in an overall yield of 88.8% containing some impurities of double protected species (20% determined from ^{1}H-NMR). The analytical data are in accordance with those reported in the literature[24].

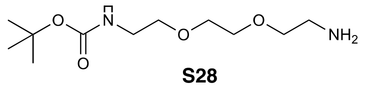

S28

^{1}H-NMR (300 MHz, $CDCl_3$): δ 5.17 (br, 1H, N*H*Boc), 3.63 (s, 4H, OCH_2CH_2O), 3.56-3.47 (m, 4H, CH_2O), 3.35-3.23 (m, 2H, CH_2NHBoc), 2.86 (t, *J* = 5.2 Hz, 2H, CH_2NH$_2$), 1.51 (s, 2H, NH_2), 1.42 (s, 9H, CH_3); ^{13}C-NMR (75 MHz, $CDCl_3$): δ 155.90, 79.03, 73.27, 70.08, 41.61, 40.21, 28.30.

N-Boc-*N'*-D-biotinyl-3,6-dioxaoctane,1,8-diamine (**S29**)

To a solution of Boc-diamine **S28** (109 mg, 0.44 mmol) and NEt_3 (81 µL, 0.59 mmol) in dry DMF (5 mL), D-biotin *N*-hydroxysuccinimide ester (S5, 100 mg, 0.29 mmol) was added and stirred for 12 h. The solvent was removed, the residue resolved in CH_2Cl_2 (40 mL), washed with 20 mL brine, dried over $MgSO_4$ and concentrated. Flash column chromatography (silica gel, CH_2Cl_2 / MeOH: 99 / 1 ➔ 93 / 7) gave compound **S29** (0.128 g, 93%). Analytical data matched the literature[25].

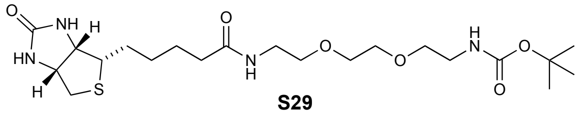

S29

TLC (CH_2Cl_2:MeOH, 90:10 v/v): R_f = 0.37; ^{1}H-NMR (300 MHz, $CDCl_3$): δ 7.35-7.23 (br, 1H. CON*H*), 6.77-6.19 (br, 2H, N*H*), 5.24-5.04 (br, 1H, CON*H*), 4.59-4.48 (m, 1H, C*H*), 4.40-4.28 (m, 1H,C*H*), 3.63 (s, 4H, OCH_2CH_2O), 3.58 (dt, $J_1 = J_2$ = 5.4 Hz, 4H, CH_2O), 3.46 (dt, $J_1 = J_2$ =4.9 Hz, 2H, CH_2NH), 3.32 (dt, $J_1 = J_2$ = 5.4 Hz, 2H, CH_2NHboc), 3.22-3.12 (m, 1H, C*H*), 2.98-2.89 (m, 1H, $CHH_{exo}S$), 2.76 (d, J = 12.8 Hz, 1H, $CHH_{endo}S$), 2.26 (t, J = 7.4 Hz, 2H, CH_2CO), 1.82-1.61 (m, 4H, CH_2), 1.46 (s, 9H, CH_3), 1.26 (m, 2H, CH_2); ^{13}C-NMR (75 MHz, $CDCl_3$): δ 173.52, 165.20, 156.94, 79.10, 70.03 (4C), 61.78, 60.30, 55.30, 40.55, 40.31, 39.13, 35.64, 29.61, 28,35 (3C) 27.96, 25.46.

N'-Boc-aminooxyacetyl-*N*-hydroxysuccinimide ester (**S30**)

1-ethyl-3-(3-dimethylaminopropyl)carbodiimid (227 mg, 1.8 mmol) and *N*-hydroxysuccinimide (190 mg, 1.65 mmol) was added to a solution of *N*-Boc-aminooxyacetic acid (287 mg, 1.5 mmol) in dry DMF (10 mL) and stirred at ambient temperature for 12 h. The mixture was diluted by the addition of H_2O (10 mL), extracted twice with EtOAc, the organic phase dried over $MgSO_4$ and concentrated under vacuum. The yellowish liquid was used without further purification (352 mg, 81%). Analytical data matched the literature[26].

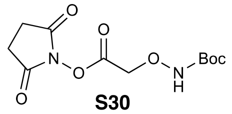

S30

^{1}H-NMR (300 MHz, $CDCl_3$): δ 7.84 (s, 1H, N*H*), 4.61 (s, 2H, CH_2), 2.82 (s, 4H, 2x CH_2), 1.31 (s, 9H, CH_3); ^{13}C-NMR (151 MHz, $CDCl_3$) δ 164.83, 162.92, 156.41, 82.17, 70.48, 27.88, 25.39.

N-aminooxyacetyl-*N'*-D-biotinyl-3,6-dioxaoctane,1,8-diamine (**S31**)

Boc protected diamine **S29** (127 mg, 0.32 mmol) was dissolved in CH_2Cl_2 (4 mL), TFA (1 mL) was added and the solution stirred at ambient temperature for 2 h. TFA was removed and the remaining solid was dried using high vacuum. The deprotected diamine was dissolved in a mixture of dry DMF (3 mL) and NEt_3 (89 µL, 0.64 mmol). Hydroxysuccinimide ester **S30** (152 mg, 0.52 mmol) was dissolved in dry DMF (0.5 mL), slowly added to the diamine and the resulting mixture was stirred at ambient temperature for 12 h. The solvent was removed and flash column chromatography (silica gel, CH_2Cl_2:MeOH, 99:1 to 93:7) gave boc protected hydroxylamine **S31** (TLC = [CH_2Cl_2:MeOH, 90:10 v/v]: R_f = 0.3). A final deprotection in 25% TFA solution (CH_2Cl_2) followed by TFA removal gave deprotected hydroxylamine S2 (102.2 mg, 71%).

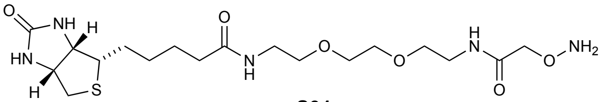

S31

^{1}H-NMR (300 MHz, D_2O): δ 4.50 (s, 2H, $COCH_2O$), 4.49-4.43 (m, 1H, C*H*), 4.24-4.31 (m, 1H,C*H*), 3.54 (s, 4H, OCH_2CH_2O), 3.52-3.45 (m, 4H, CH_2O), 3.33 (dt, $J_1 = J_2$ =5.3 Hz, 2H, CH_2NH), 3.24 (dt, $J_1 = J_2$ = 5.4 Hz, 2H, CH_2NHboc), 3.21-3.14 (m, 1H, C*H*), 2.85 (dd, J_1 = 13, J_2 = 4.9, Hz 1H, $CHH_{exo}S$), 2.62 (d, J = 13 Hz, 1H, $CHH_{endo}S$), 2.13 (t, J = 7.2 Hz, 2H, CH_2CO), 1.65-1.36 (m, 4H, CH_2), 1.32-1.20 (m, 2H, CH_2); ^{13}C-NMR (151 MHz, D_2O) δ 176.79, 168.56, 165.14, 71.52, 69.26, 69.23, 68.70, 68.44, 61.93, 60.09, 55.20, 39.52, 38.67, 38.50, 35.26, 27.68, 27.52, 24.97; ESI-MS (*m/z*):[M]$^+$ calcd. for $C_{18}H_{34}N_5O_6S$, 448.22; found 448.21.

3-(N-iminoacetyl-N'-D-biotinyl-3,6-dioxaoctane,1,8-diamine)-tyrosine (**26**)

Hydroxylamine **S31** (20 mg, 0.04 mmol) was dissolved in 1 mL NH_4OAc pH 4.5, tyrosine **S16** was added (9,34 mg 0.04 mmol) and the solution incubated at 37 °C, 200 rpm for 4 h. The reaction mixture was purified by preparative HPLC (method D). The oxime was obtained with a yield of 71% (20 mg, 0.03 mmol).

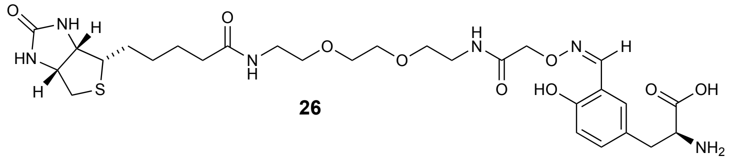

26

^{1}H-NMR (600 MHz, D_2O): δ 8.51 (s, 1H, ONCH), 7.37 (d, *J* = 2.3 Hz, 1H, CH_{phenyl}), 7.31 (dd, *J* = 8.4, 2.3 Hz, 1H, CH_{phenyl}), 7.01 (d, *J* = 8.4 Hz, 1H, CH_{phenyl}), 4.72 (s, 2H, $COCH_2O$), 4,57 (dd, *J* = 7.9, 4.9 Hz, 1H, CH), 4.37 (dd, *J* = 7.9, 4.5 Hz, 1H,CH), 4.28 (dd, *J* = 7.4, 5.8 Hz, 1H, CH), 3.65-3.59 (m, 4H OCH_2CH_2O), 3.53-3.46 (m, 6H, CH_2O, CH_2NH), 3.32-3.26 (m, 5H, CH_2NHboc, CH, CH_2), 3.19 (dd, *J* = 14.8, 7.4 Hz 1H, $CHH_{exo}S$), 2.96 (d, *J* = 13, 5.0 Hz, 1H, $CHH_{endo}S$), 2.23 (t, *J* = 7.3 Hz, 2H, CH_2CO), 1.71-1.50 (m, 4H, CH_2), 1.42-1.33 (m, 2H, CH_2); ^{13}C-NMR (151 MHz, D_2O) δ, 176.81, 172.01, 171.58, 165.30, 155.30, 152.38, 133.16, 131.05, 126.10, 117.30, 117.13, 116.84, 72.54, 69.57, 69.44, 68.87, 68.83, 62.08, 60.25, 55.36, 54.25, 39.69, 38.83, 35.45, 34.74, 27.88, 27.68, 25.13; ESI-MS (*m/z*):[M]$^+$ calcd. for $C_{18}H_{34}N_5O_6S$, 638.73; found 638.72.

2.4 Synthesis of CF–Tub-tag peptide 3

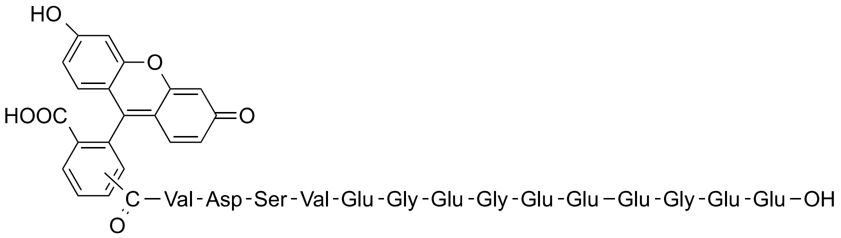

CF–Tub-tag peptide was synthesized by standard Fmoc-based chemistry in a linear synthesis on an Activotec peptide synthesizer followed by manual coupling of 5(6)-carboxyfluorescein. 0.1 mmol of Fmoc-*L*-Glu(tBu)-Wang resin (subst: 0.58 mmol/g) was added to a reaction vessel and synthesis was performed with five fold amino acid excess. Coupling was achieved by HOBt/HBTU/DIPEA addition. After the final amino acid coupling, the fluorophore was coupled in a double coupling procedure with 5 eq of 5(6)-carboxyfluorescein, HOBt, HBTU and DIPEA in DMF for 1 h. The peptide was cleaved off the resin by addition of TFA/DTT/Tis/thioanisol (95/2/2/1) within 4 h. Subsequently, the cleavage cocktail was evaporated by N_2-flow and the peptide was precipitated by the addition of ice-cold diethyl ether. The precipitate was spun down, dissolved in water and purified by preparative HPLC (method D). The peptide was obtained with a yield of 8% (16 mg, 8 µmol); molar mass peptide = 1850.6 Da; ESI-HRMS (*m/z*): $[M+2H]^{2+}$ calcd. 926.3165; found 926.3065.

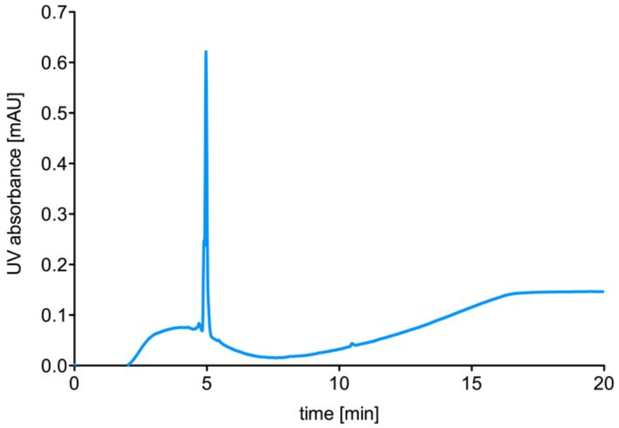

Supplementary Figure 15.LC-UV at 220 nm, 10 to 100% of acetonitrile in water containing 0.1% TFA on a RP-C18 column.

3. NMR Spectra of S3, 7, 24 and 26

3.1 3-formyl-*L*-tyrosine (S16)

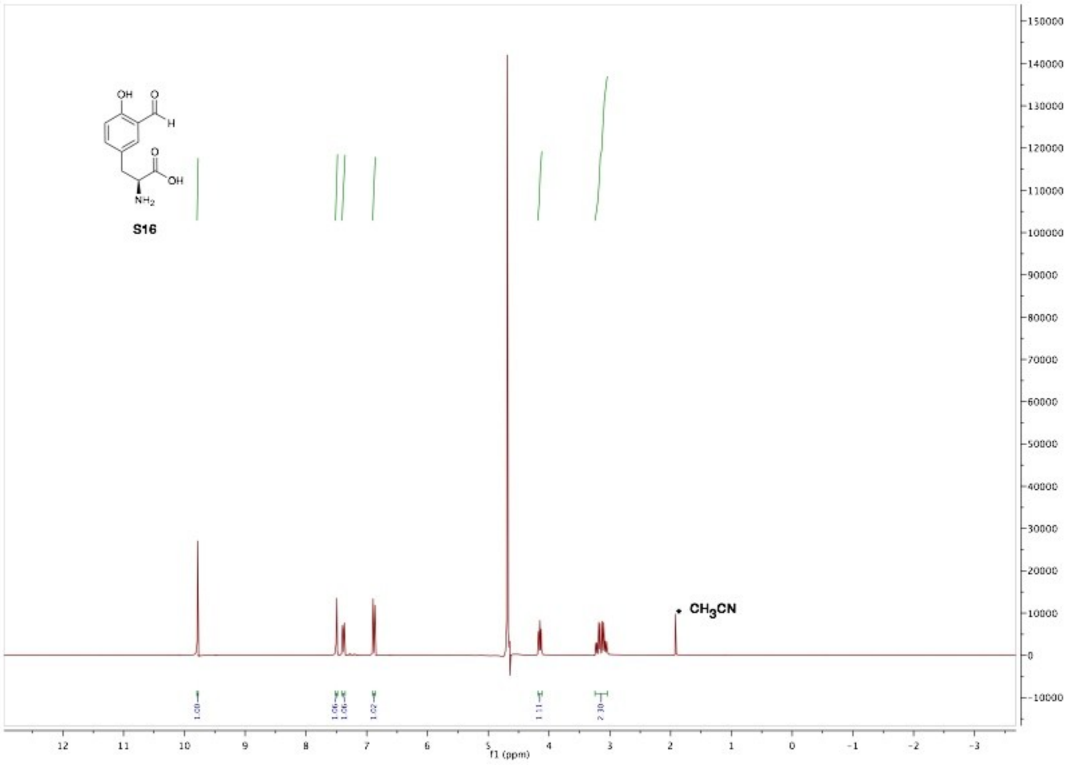

Supplementary Figure 16. ^{1}H-NMR (300 MHz, D_2O) of 3-formyl-*L*-tyrosine (**S16**)

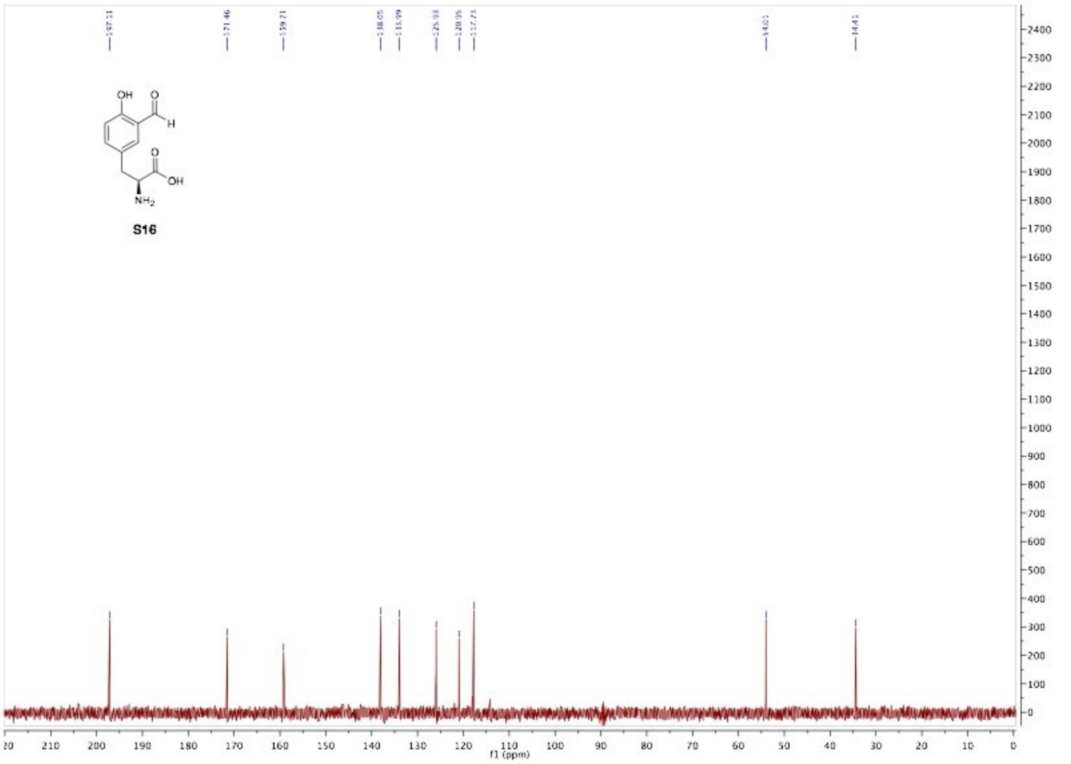

Supplementary Figure 17. ^{13}C-NMR (75 MHz, D_2O) of 3-formyl-*L*-tyrosine (**S16**)

3.5 Tyr(*o*-propargyl) 7

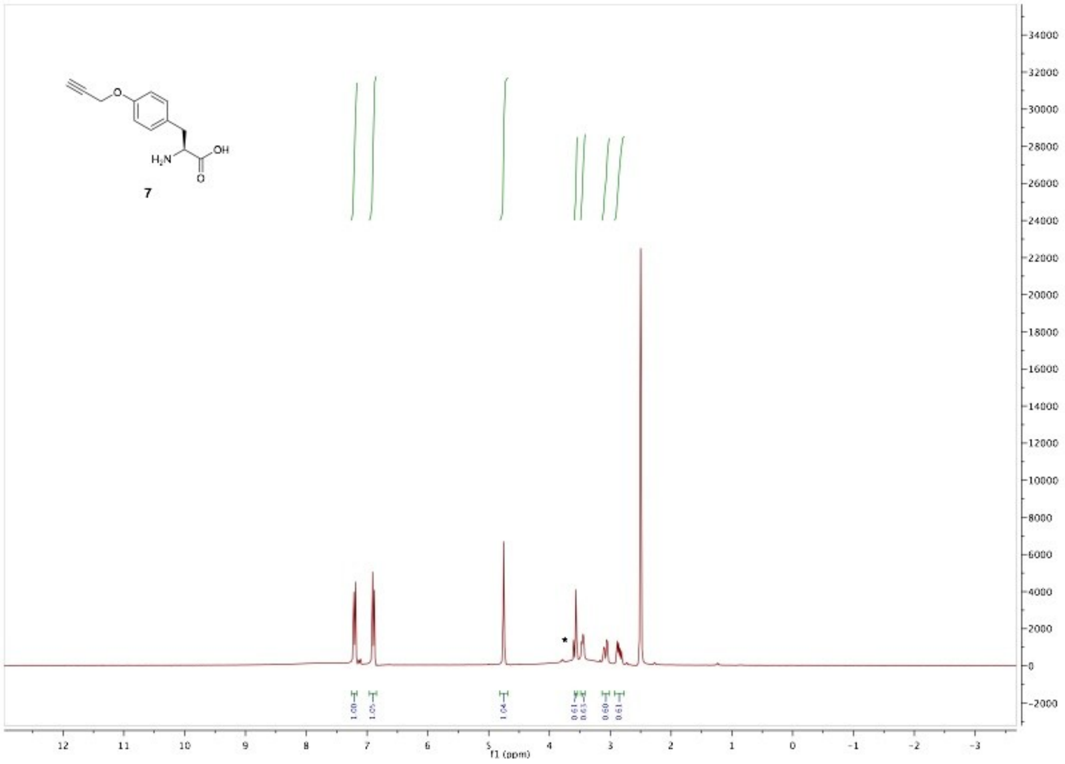

Supplementary Figure 18. ^{1}H-NMR (151 MHz, D_2O) of **7**

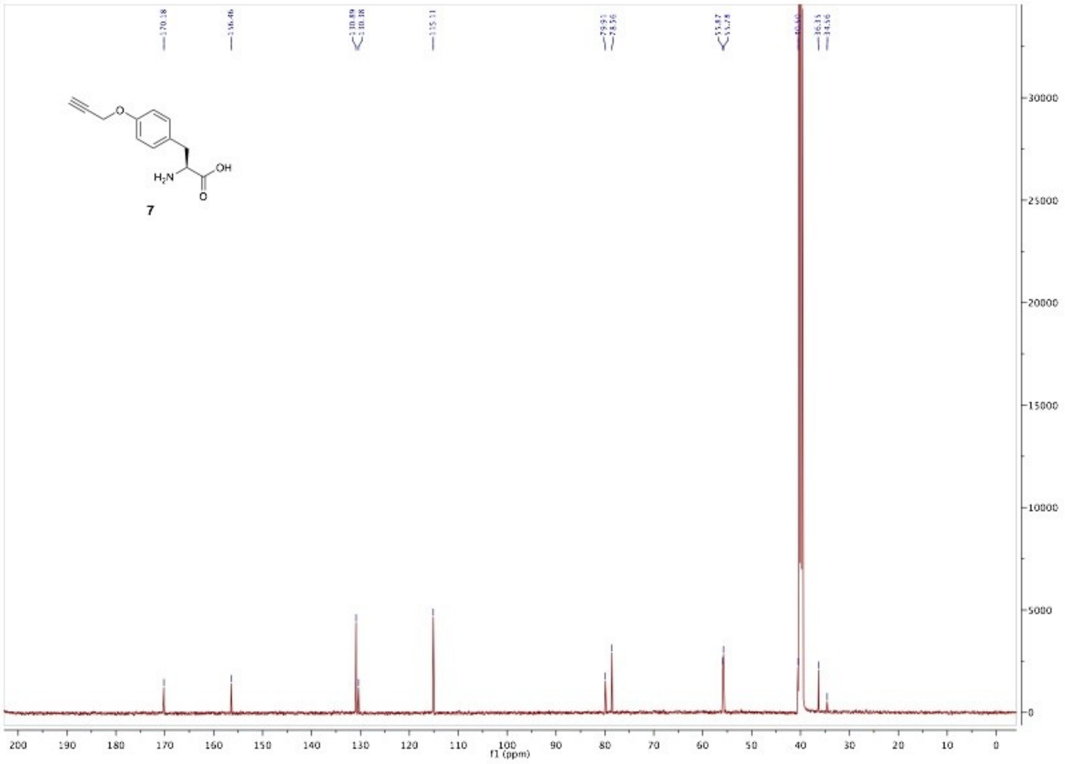

Supplementary Figure 19. ^{13}C-NMR (151 MHz, DMSO-d_6) of **7**

3.5 Coumarin 24

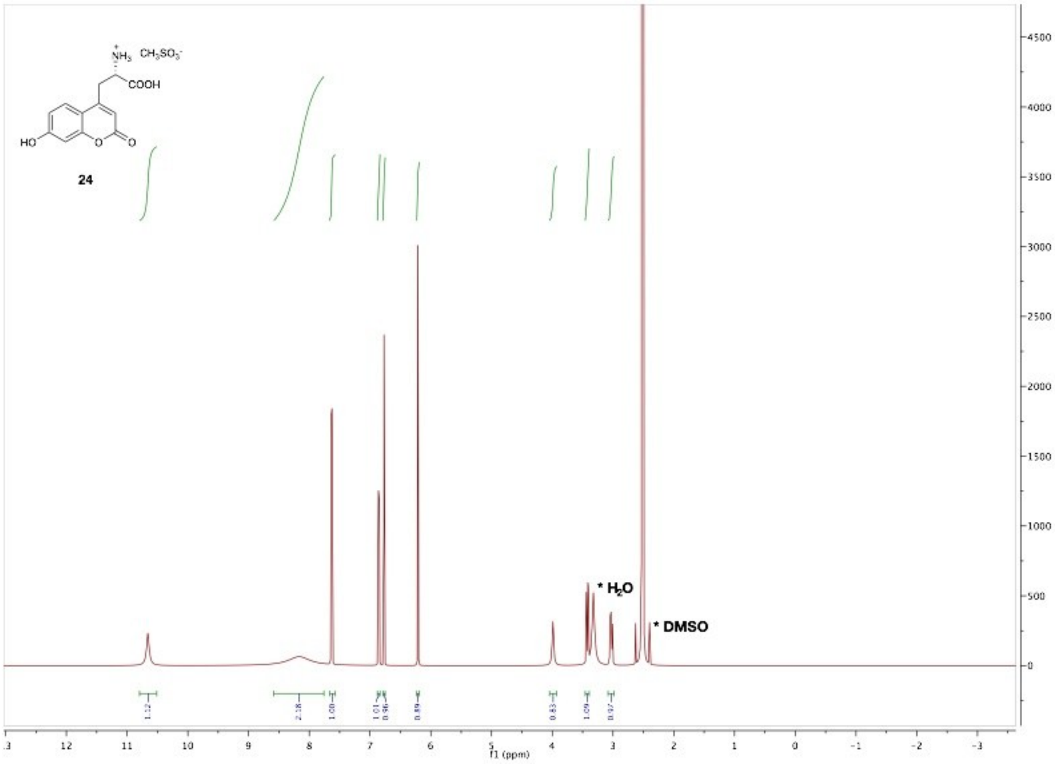

Supplementary Figure 20. ^{1}H-NMR (600 MHz, DMSO-d_6) of Coumarin **24**

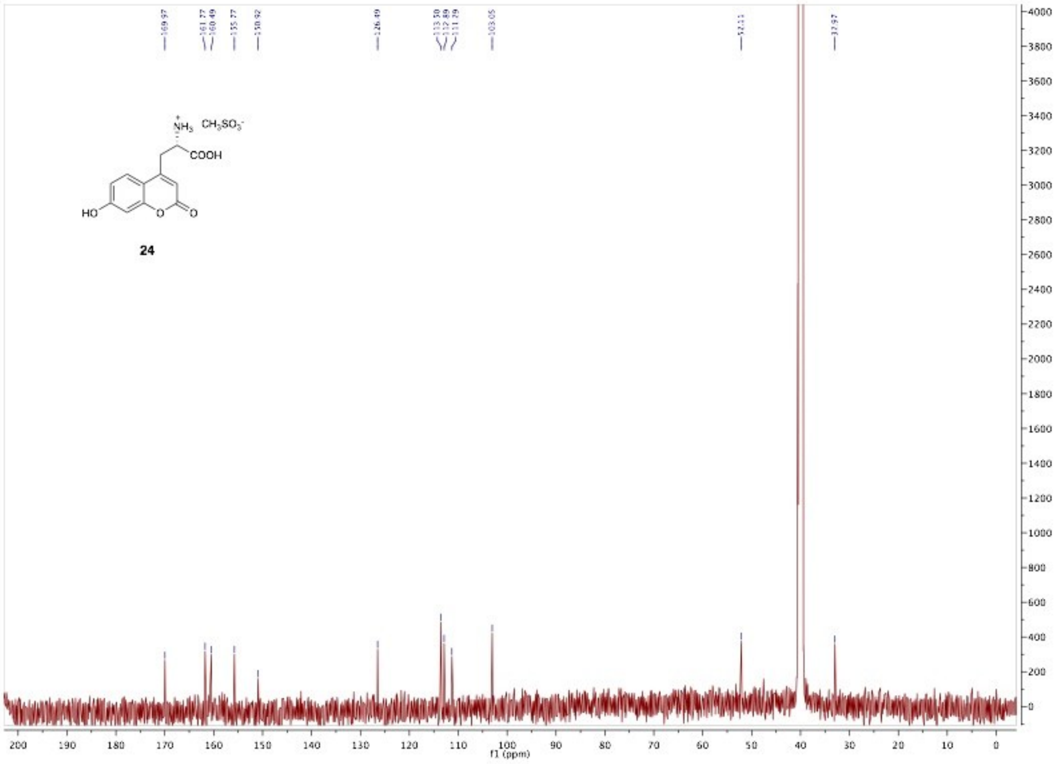

Supplementary Figure 21. ^{13}C-NMR (151 MHz, DMSO-d_6) of Coumarin **24**

3.5 Tyrosine biotin 26

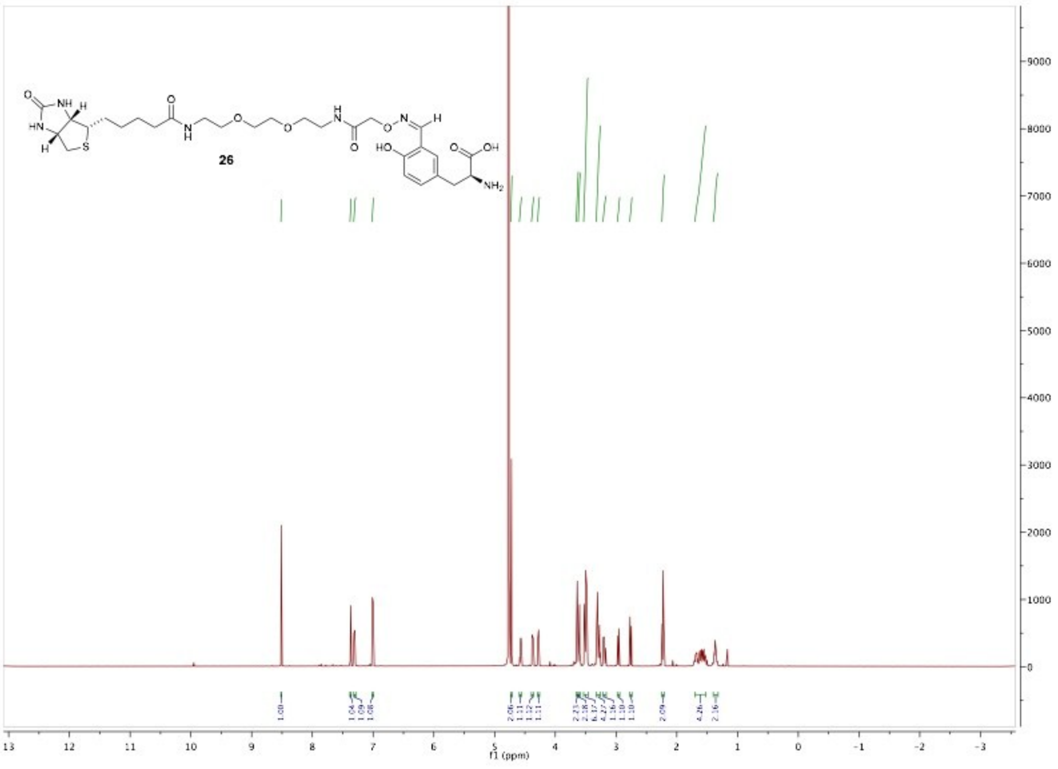

Supplementary Figure 22. ^{1}H-NMR (600 MHz, D_2O) of biotin **26**

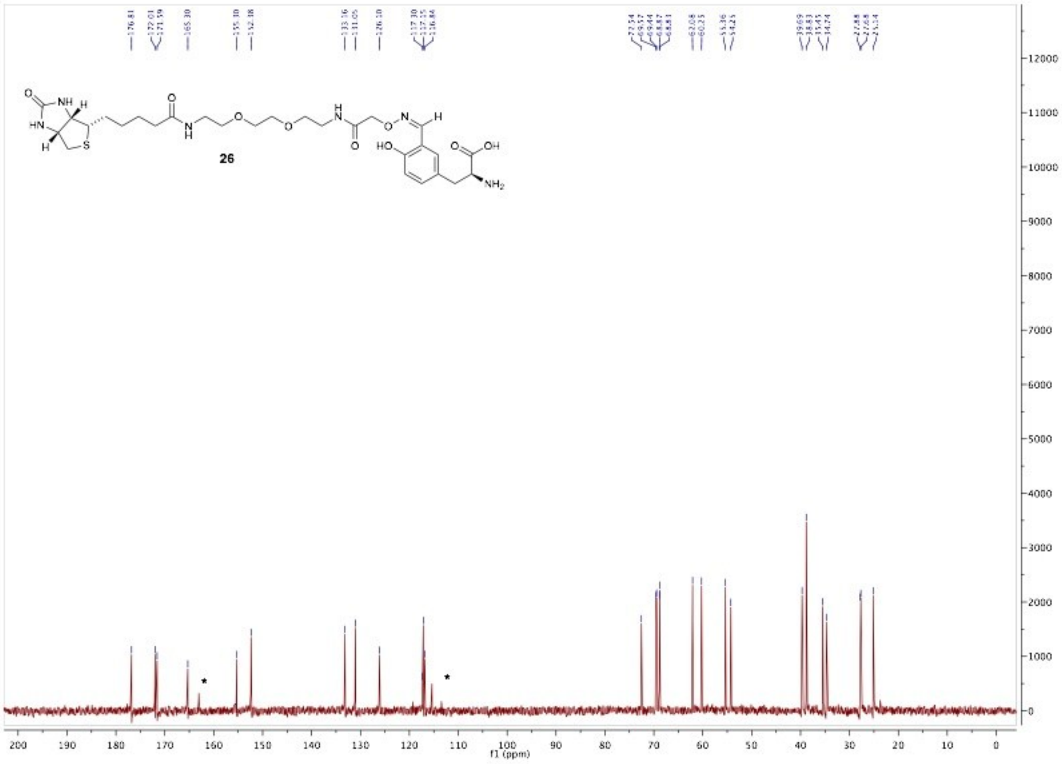

Supplementary Figure 23. ^{13}C-NMR (151 MHz, D_2O) of biotin **26**

4. References

1. D. Schumacher, J. Helma, F. A. Mann, G. Pichler, F. Natale, E. Krause, M. C. Cardoso, C. P. Hackenberger and H. Leonhardt, *Angew. Chem. Int. Ed.*, 2015, **54**, 13787-13791.
2. S. Zink, L. Grosse, A. Freikamp, S. Banfer, F. Muksch and R. Jacob, *J. Cell. Sci.*, 2012, **125**, 5998-6008.
3. H. Leonhardt, H. P. Rahn, P. Weinzierl, A. Sporbert, T. Cremer, D. Zink and M. C. Cardoso, *J. Cell. Biol.*, 2000, **149**, 271-280.
4. H. P. Easwaran, L. Schermelleh, H. Leonhardt and M. C. Cardoso, *EMBO Rep.*, 2004, **5**, 1181-1186.
5. N. Daigle, J. Beaudouin, L. Hartnell, G. Imreh, E. Hallberg, J. Lippincott-Schwartz and J. Ellenberg, *J. Cell. Biol.*, 2001, **154**, 71-84.
6. G. M. Morris, R. Huey, W. Lindstrom, M. F. Sanner, R. K. Belew, D. S. Goodsell and A. J. Olson, *J. Comput. Chem.*, 2009, **30**, 2785-2791.
7. A. E. Prota, M. M. Magiera, M. Kuijpers, K. Bargsten, D. Frey, M. Wieser, R. Jaussi, C. C. Hoogenraad, R. A. Kammerer, C. Janke and M. O. Steinmetz, *J. Cell Biol.*, 2013, **200**, 259-270.
8. G. M. Morris, D. S. Goodsell, R. S. Halliday, R. Huey, W. E. Hart, R. K. Belew and A. J. Olson, *J. Comput. Chem.*, 1998, **19**, 1639-1662.
9. D. Van Der Spoel, E. Lindahl, B. Hess, G. Groenhof, A. E. Mark and H. J. Berendsen, *J. Comput. Chem.*, 2005, **26**, 1701-1718.
10. K. Lindorff-Larsen, S. Piana, K. Palmo, P. Maragakis, J. L. Klepeis, R. O. Dror and D. E. Shaw, *Proteins*, 2010, **78**, 1950-1958.
11. W. L. Jorgensen, J. Chandrasekhar, J. D. Madura, R. W. Impey and M. L. Klein, *J. Chem. Phys.*, 1983, 926-935.
12. D. A. Case, R. M. Betz, W. Botello-Smith, D. S. Cerutti, T. E. Cheatham, T. A. Darden, R. E. Duke, T. J. Giese, H. Gohlke, A. W. Goetz, N. Homeyer, S. Izadi, P. Janowski, J. Kaus, A. Kovalenko, T. S. Lee, S. LeGrand, P. Li, C. Lin, T. Luchko, R. Luo, B. Madej, D. Mermelstein, K. M. Merz, G. Monard, H. Nguyen, H. T. Nguyen, I. Omelyan, A. Onufriev, D. R. Roe, A. Roitberg, C. Sagui, C. L. Simmerling, J. Swails, R. C. Walker, J. Wang, R. M. Wolf, X. Wu, L. Xiao, D. M. York and P. A. Kollman, *Amber 2016*, 2016.
13. A. W. Sousa da Silva and W. F. Vranken, *BMC Res. Notes.*, 2012, **5**, 367.
14. G. Bussi, D. Donadio and M. Parrinello, *J. Chem. Phys.*, 2007, **126**, 014101.
15. B. Hess, H. Bekker, H. J. Berendsen and J. G. E. M. Fraaije, *J. Comput. Chem.*, 1997, **18**, 1463-1472.
16. T. Darden, D. York and L. Pedersen, *J. Chem. Phys.*, 1993, **98**, 10089-10092.
17. M. E. Jung and T. I. Lazarova, *J. Org. Chem.*, 1997, **62**, 1553-1555.
18. A. Banerjee, T. D. Panosian, K. Mukherjee, R. Ravindra, S. Gal, D. L. Sackett and S. Bane, *ACS Chem. Biol.*, 2010, **5**, 777-785.
19. S. Milles, S. Tyagi, N. Banterle, C. Koehler, V. VanDelinder, T. Plass, A. P. Neal and E. A. Lemke, *J. Am. Chem. Soc.*, 2012, **134**, 5187-5195.
20. M. P. Brun, L. Bischoff and C. Garbay, *Angew. Chem. Int. Ed.*, 2004, **43**, 3432-3436.
21. K. Hafner and K. P. Meinhardt, *Org. Synth.*, 1984, **62**, 134.
22. G. Loidl, H. J. Musiol, N. Budisa, R. Huber, S. Poirot, D. Fourmy and L. Moroder, *J. Pept. Sci.*, 2000, **6**, 139-144.
23. E. Gerard, A. Meulle, O. Feron and J. Marchand-Brynaert, *Bioorganic & medicinal chemistry letters*, 2012, **22**, 586-590.
24. M. Ishida, H. Watanabe, K. Takigawa, Y. Kurishita, C. Oki, A. Nakamura, I. Hamachi and S. Tsukiji, *J Am Chem Soc*, 2013, **135**, 12684-12689.

25. M. Braun, X. Camps, O. Vostrowsky, A. Hirsch, E. Endreß, T. M. Bayerl, O. Birkert and G. Gauglitz, *Eur. J. Org. Chem.*, 2000, **7**, 1173.
26. K. K. Palaniappan, R. M. Ramirez, V. S. Bajaj, D. E. Wemmer, A. Pines and M. B. Francis, *Angew. Chem. Int. Ed.*, 2013, **52**, 4849-4853.
